# Accounting for the Competing Risk of Death to Predict Kidney Failure in Adults With Stage 4 Chronic Kidney Disease

**DOI:** 10.1001/jamanetworkopen.2021.9225

**Published:** 2021-05-04

**Authors:** Huda Al-Wahsh, Navdeep Tangri, Rob Quinn, Ping Liu, Thomas Ferguson, MS, Marta Fiocco, Ngan N. Lam, MD, MSc, Marcello Tonelli, Pietro Ravani

**Affiliations:** 1 Cumming School of Medicine, Department of Medicine, University of Calgary, Calgary, Alberta, Canada; 2 Department of Medicine, Department of Community Health Sciences, Seven Oaks General Hospital, University of Manitoba, Winnipeg, Manitoba, Canada; 3 Mathematical Institute, Leiden University, Medical Statistics Section, Department of Biomedical Data Science, Leiden University Medical Centre, Leiden, the Netherlands

## Abstract

**Importance:**

Kidney failure risk prediction has implications for disease management, including advance care planning in adults with severe (ie, estimated glomerular filtration rate [eGFR] category 4, [G4]) chronic kidney disease (G4-CKD). Existing prediction tools do not account for the competing risk of death.

**Objective:**

To compare predictions of kidney failure (defined as estimated glomerular filtration rate [eGFR] <10 mL/min/1.73 m^2^ or initiation of kidney replacement therapy) from models that do and do not account for the competing risk of death in adults with G4-CKD.

**Design, Setting, and Participants:**

This prognostic study linked population-based laboratory and administrative data (2002-2017) from 2 Canadian provinces (Alberta and Manitoba) to compare 3 kidney risk models: the standard Cox regression, cause-specific Cox regression, and Fine-Gray subdistribution hazard model. Participants were adults with incident G4-CKD (eGFR 15-29 mL/min/1.73 m^2^). Data analysis occurred between July and December 2020.

**Main Outcomes and Measures:**

The performance of kidney risk models at prespecified times and across categories of baseline characteristics, using calibration, reclassification, and discrimination (for competing risks). Predictive characteristics were age, sex, albuminuria, eGFR, diabetes, and cardiovascular disease.

**Results:**

The development and validation cohorts included 14 619 (7070 [48.4%] men; mean [SD] age, 74.1 [12.8] years) and 2295 (1152 [50.2] men; mean [SD] age, 71.9 [14.0] years) adults, respectively. The 3 models had comparable calibration up to 2 years from entry. Beyond 2 years, the standard Cox regression overestimated the risk of kidney failure. At 4 years, for example, risks predicted from standard Cox were 40% for people whose observed risks were less than 30%. At 2 years (risk cutoffs 10%-20%) and 5 years (risk cutoffs 15%-30%), 788 (5.4%) and 2162 (14.8%) people in the development cohort were correctly reclassified into lower- or higher-risk categories by the Fine-Gray model and incorrectly reclassified by standard Cox regression (the opposite was observed in 272 patients [1.9%] and 0 patients, respectively). In the validation cohort, 115 (5.0%) individuals and 389 (16.9%) individuals at 2 and 5 years, respectively, were correctly reclassified into lower- or higher-risk categories by the Fine-Gray model and incorrectly reclassified by the standard Cox regression; the opposite was observed in 98 (4.3%) individuals and 0 individuals, respectively. Differences in discrimination emerged at 4 to 5 years in the development cohort and at 1 to 2 years in the validation cohort (0.85 vs 0.86 and 0.78 vs 0.8, respectively). Performance differences were minimal during the entire follow-up in people at lower risk of death (ie, aged ≤65 years or without cardiovascular disease or diabetes) and greater in those with a higher risk of death. At 5 years, for example, in people aged 65 years or older, predicted risks from standard Cox were 50% where observed risks were less than 30%. Similar miscalibration was observed at 5 years in people with albuminuria greater than 30 mg/mmol, diabetes, or cardiovascular disease.

**Conclusions and Relevance:**

In this study, predictions about the risk of kidney failure were minimally affected by consideration of competing risks during the first 2 years after developing G4-CKD. However, traditional methods increasingly overestimated the risk of kidney failure with longer follow-up time, especially among older patients and those with more comorbidity.

## Key Points

**Question:**

Does accounting for the competing risk of death make a difference when predicting kidney failure in adults with stage 4 chronic kidney disease?

**Findings:**

In this external prognostic study with 14 619 people in the development cohort and 2295 in the validation cohort, models that did and did not account for the competing risk of death provided comparable 2-year predictions of kidney failure. Differences in model predictions emerged after 2 years and increased with longer prediction times, especially among participants aged 65 years or older and those with more comorbidity.

**Meaning:**

These findings suggest that accounting for the competing risk of death when making predictions about kidney failure becomes increasingly important with longer follow-up time, older age, and the presence of more comorbidities.

## Teaser

This population-based validation study compares the prediction of kidney failure from models that do and do not account for the competing risk of death in adults with severe chronic kidney disease.

## Introduction

Chronic kidney disease (CKD) is defined by an estimated glomerular filtration rate (eGFR) less than 60 mL/min/1.73 m^2^ and affects 10% to 16% of the general population globally.^1^ CKD is associated with increasing risk of morbidity and mortality as kidney function declines. For this reason, people with severe (ie, eGFR category 4, [G4]) CKD (ie, an eGFR of 15-29 mL/min/1.73 m^2^) and a high predicted risk of kidney failure are advised to discuss treatment options for kidney failure, including kidney replacement therapy (ie, dialysis or kidney transplantation) or conservative (ie, palliative) care. However, most patients with G4-CKD are 70 years of age or older and are 3 to 10 times more likely to die than develop kidney failure.^2^ Therefore, accounting for the competing risk of death could be an important consideration for clinical decision-making.

Kidney failure risk calculators are routinely used in clinical practice to risk-stratify patients and inform nephrology referral or treatment decisions.^3,4^ These tools were developed using methods that do not account for the competing risk of death and, thus, overestimate the risk of kidney failure.^5,6^ Censoring for a competing event is assumed to leave the risk of the event of interest unaltered, but the risk of kidney failure becomes 0 after death, and many people with CKD die without kidney failure.^2,6^ The extent of this bias is unknown, especially in short-term predictions when risk overestimation because of censoring for competing risks may be minimal. Understanding how failing to account for the competing risk of death may affect the predictions made by such kidney failure risk calculators is important because they are already implemented in many electronic clinical support systems.

In this population-based study, we identified adults with incident G4-CKD and examined the prognostic performance of standard Cox regression with 2 methods that account for competing risks—the cause-specific Cox regression and the Fine-Gray model. We developed and assessed the performance of models using data from 2 separate CKD cohorts at prespecified times and across categories of baseline characteristics to determine when, and in whom, differences between models that do and do not account for competing risks are most pronounced.

## Methods

### Study Design and Data Sources

Both the development cohort (from Alberta, Canada) and the validation cohort (from Manitoba, Canada) for this prognostic study were formed using population-based, linked, provincial administrative and laboratory data. The institutional ethics review boards at the University of Alberta, University of Calgary, and University of Manitoba approved this study with a waiver of participant consent because retrospective, deidentified data were used. We followed the Reporting of Studies Conducted Using Observational Routinely Collected Data (RECORD) reporting guideline and the Transparent Reporting of a Multivariable Prediction Model for Individual Prognosis or Diagnosis (TRIPOD) reporting guideline.^7,8^

### Population

We identified 2 provincial cohorts of residents from Alberta and Manitoba who were at least 18 years old and had G4-CKD (ie, an eGFR of 15-29 mL/min/1.73 m^2^). We calculated eGFR using the chronic kidney disease epidemiology equation, with serum creatinine values standardized to isotope dilution mass spectrometry traceable methods.^9^ Given the lack of information on race and the low prevalence of people who self-identify as Black in Alberta (3.3%)^10^ and Manitoba (2.4%),^11^ we calculated eGFR assuming that all participants were White, as in our previous work.^5^ We applied a moving average eGFR method to identify newly documented G4-CKD cases using outpatient laboratory measurements between July 30, 2002, and March 31, 2014, for the development cohort, and between April 1, 2008, and March 31, 2014, for the validation cohort. This method minimizes the inclusion of people with acute kidney injury, unstable clinical conditions, or preexisting G4-CKD or kidney failure.^12^ We determined the mean eGFR calculated using repeated measures within an individual recorded over a period of at least 90 days starting on the date of the first eGFR of less than 30 mL/min/1.73 m^2^, provided that there were at least 2 measurements to calculate the mean. In addition, the first and the last eGFR were separated by more than 90 days, and all intervening measurements were within 90 days. We used the minimum value of eGFR when there were multiple measurements on the same day. Participants met the study entry criterion if the mean eGFR (index eGFR) during this period was 15 to 30 mL/min/1.73 m^2^. We used the date of the last eGFR measurement included in the calculation of the mean eGFR to define cohort entry (index date). We excluded patients who had received kidney replacement therapy or had an eGFR of less than 15 mL/min/1.73 m^2^ before the index date. We also excluded people without information on albuminuria because this variable has been included in all existing kidney failure risk calculators.^3,4^

### Independent Variables

We incorporated baseline covariates that are known to be associated with kidney failure in the models ^3,4^: age, sex, diabetes, cardiovascular disease, index eGFR, and albuminuria. We identified comorbid conditions using the *International Classification of Diseases, Ninth Revision, Clinical Modification* (*ICD-9-CM*) and *ICD-10* applied to physician claims and hospitalization data prior study entry (eTable 1 in the Supplement).^13^ We defined baseline albuminuria as the most recent albuminuria value on or within the 2 years preceding the index date.

### Outcomes and Follow-up Time

The outcome was kidney failure,^2,5^ which we defined as the earliest initiation of kidney replacement therapy (dialysis or kidney transplantation) or having an eGFR of less than 10 mL/min/1.73 m^2^. Dialysis initiation was ascertained by at least 1 inpatient or outpatient physician claim and from the provincial registry of chronic dialysis. The receipt of a kidney transplant was based on at least 1 physician claim or hospitalization (eTable 2 in the Supplement). For the eGFR criterion, we applied the same moving average method we used to define cohort entry starting on the date of the first inpatient or outpatient eGFR of less than 10 mL/min/1.73 m^2^ over a period of more than 90 days and provided that there were at least 2 measurements.^12^ The event date was defined as the date of the last eGFR measured during this period. In both cohorts, patients were followed from the date of cohort entry until kidney failure, death, study end (March 31, 2017), emigration from the province, or 10 years from cohort entry. To minimize bias in outcome ascertainment, we censored observations at 1.5 years from an eGFR if no subsequent measurement was available within 1.5 years of this eGFR, as in previous studies.^2,5^

### Statistical Analysis

We estimated crude risks using the Kaplan-Meier method, which censors for competing events, and the Aalen-Johansen method, which accounts for competing events. We developed and validated 3 semiparametric models for the development and validation cohort, respectively. The standard Cox regression of kidney failure treated death as a noninformative censoring event, ie, an event that does not alter the risk of the event of interest.^14^ The model assumes a 1-to-1 relationship between risk of the event of interest at a certain point in time and the cumulative hazard for that event. We used the following 2 models to account for the competing risk of death: the cause-specific hazard model of kidney failure and death and the Fine-Gray subdistribution hazard model. In the cause-specific hazard model, the standard Cox regression is used to model each competing event, and persons who move to another disease state (ie, death for the kidney failure model and kidney failure for the death model) are censored at their transition time. The cumulative incidence function for a specific cause *k* not only depends on the hazard of cause *k*, but also on the hazards of all other causes,^15^ and is obtained indirectly from the model parameters.^16,17^ The Fine-Gray model of kidney failure estimates the covariate effects on the cumulative incidence function of kidney failure in terms of subhazard ratios and links the cumulative incidence function to the subhazard function of kidney failure directly.^18,19^

We included age, sex, eGFR, albuminuria, diabetes, and presence of cardiovascular disease in all models and tested all possible first-order interactions among these variables, retaining interaction terms that improved the goodness-of-fit of the model. We used Martingale residuals to assess the association between continuous covariates and outcome considering the use of quadratic terms or log-transformations to model nonlinear relationships. We used residual analyses to identify deviations from the proportionality assumption, influential observations, and outliers and assess the goodness-of-fit.^20,21^ During model building, we checked that the results were consistent across study time.

We assessed model performance using different methods. In calibration analysis, we evaluated the graphical agreement between observed and predicted risks at 1 to 8 years. In an ideal model, pairs of the observed and predicted risks lie on a 45° angle line. In reclassification analysis, we used the cutoffs at 2 years of 0% to less than 10%, 10% to less than 20%, and 20% or more at 2 years, and at 5 years of 0% to less than 15%, 15% to less than 30%, and 30% or more to define low-, intermediate-, and high-risk categories of kidney failure. These cutoffs were used in previous studies^3^ or existing recommendations (10% is the 2-year risk threshold used for multidisciplinary kidney care referral).^22^ We obtained individual risk predictions and populated a 3 × 3 table according to the predictions of rival models. We calculated the observed risk of the members assigned to each cell using the Aalen-Johansen function and obtained the total number and percentage of people who were incorrectly and correctly classified by each model. This approach addresses some limitations with net reclassification^23^ and has been proposed for the competing risks settings.^24^ We also used model concordance (C statistic) with methods for competing risks to assess the model's ability to separate individuals with kidney failure from those without kidney failure, ranging from 0.5 (no prediction ability beyond chance) to 1 (perfect discrimination).^23,25,26^ In general, a C index of 0.5 suggests no discrimination, 0.7 to 0.8 is considered acceptable, 0.8 to 0.9 is considered excellent, and more than 0.9 is considered outstanding.^27^ We also plotted time-dependent Brier scores accounting for censoring. Smaller scores indicated better performance in terms of both discrimination and calibration.^28^

We repeated these analyses across categories defined by the following baseline characteristics: patients 65 years or younger vs patients older than 65 years, sex, albumin-to-creatinine ratio of 30 mg/mmol or less vs greater than 30 mg/mmol (to convert albumin to grams per liter, multiply by 10; creatinine to micromoles per liter, multiply by 88.4), eGFR of 27 or less vs greater than 27 mL/min/1.73 m^2^ (the median eGFR in previous studies),^2,5^ diabetes status, and cardiovascular disease. We also repeated the reclassification analysis considering different thresholds,^29^ including those used in general practice for nephrology referral (5% at 5 years) or preparation for dialysis (40% at 2 years).^30^ In 1 sensitivity analysis, we assessed the consistency of results in subgroups defined by index date on or before vs after the median. In another sensitivity analysis, we defined kidney failure by the initiation of kidney replacement or the occurrence of sustained (as opposed to moving average) eGFR of less than 10 mL/min/1.73 m^2^. We defined sustained eGFR of less than 10 mL/min/1.73 m^2^ by the occurrence of 2 or more consecutive eGFR values of less than 10 mL/min/1.73 m^2^ for more than 90 days. We used the date of the last eGFR measurement as the event date. We used the packages prodlim, cmprsk, and riskRegression in R version 4.0.3 for all analyses (R Project for Statistical Computing). We performed all analyses between July and December 2020.

## Results

### Cohort Description

We identified 14 619 people who met the study eligibility criteria in the development and 2295 who met the eligibility criteria in the validation cohorts (eFigure 1 in the Supplement). The 2 cohorts had a comparable distribution of sex and index eGFR (7070 men [48.4%] in the development cohort and 1152 men [50.2%] in the validation cohort) and index eGFR (median [IQR] 27.6 [25.1-29] mL/min/1.73 m^2^ vs 25 [22-27] mL/min/1.73 m^2^). The development cohort was slightly older than the validation cohort (mean [SD] age, 74.1 [12.8] years vs 71.9 [14] years), had lower albuminuria (median [IQR] albumin-creatinine ratio, 7.6 [1.7-56.2] mg/mmol vs 19.7 [3.3-136.1] mg/mmol), and included more people with diabetes (9886 [67.6%] vs 1448 [63.1%]) and cardiovascular disease (8285 [56.7%] vs 796 [34.7%]) (Table). Baseline characteristics of the development cohort (which had longer accrual time) did not change over time (eTable 3 in the Supplement). The incidence rate of kidney failure was lower in the development cohort than in the validation cohort, while mortality rates were similar in both cohorts (incidence rate of kidney failure, 6.1 [95% CI, 5.9-6.4] events per 100 person-years vs 10.3 [95% CI, 9.5-11.0] events per 100 person-years; mortality rate, 12.3 [95% CI, 12.0-12.6] events per 100 person-years vs 12.4 [95% CI, 11.6-13.3] events per 100 person-years).

**Table.** Baseline Characteristics of Participants in the Development and Validation Cohorts

Characteristic	No. (%)	
	Development cohort (n = 14 619)	Validation cohort (n = 2295)
Age, mean (SD), y	74.1 (12.8)	71.9 (14.0)
Men	7070 (48.4)	1152 (50.2)
Women	7549 (51.6)	1143 (49.8)
eGFR, median (IQR), mL/min/1.73 m^2^	27.6 (25.1-29.0)	25.0 (22.0-27.0)
ACR, median (IQR), mg/mmol	7.6 (1.7-56.2)	19.7 (3.3-136.1)
Diabetes	9886 (67.6)	1448 (63.1)
Cardiovascular disease		
Any	8285 (56.7)	796 (34.7)
Myocardial infarction	1791 (12.3)	147 (6.4)
Congestive heart failure	5735 (39.2)	602 (26.2)
Stroke or TIA	3594 (24.6)	213 (9.3)
Peripheral vascular disease	1217 (8.3)	52 (2.3)
Follow-up time, median (IQR), y	3.2 (1.5-5.2)	2.9 (1.3-4.3)
Competing events		
Kidney failure	3265 (22.3)	722 (37.5)
Incidence per 100 person-years (95% CI)	6.1 (5.9-6.4)	10.3 (9.5-11.0)
Death without kidney failure	6528 (44.7)	875 (38.1)
Incidence per 100 person-years (95% CI)	12.3 (12.0-12.6)	12.4 (11.6-13.3)

Abbreviations: ACR, albumin-to-creatinine ratio; eGFR, estimated glomerular filtration rate; IQR, interquartile range; TIA, transient ischemic attack.

SI conversions: To convert albumin to grams per liter, multiply by 10; creatinine to micromoles per liter, multiply by 88.4.

### Crude Risks

**Figure 1.**
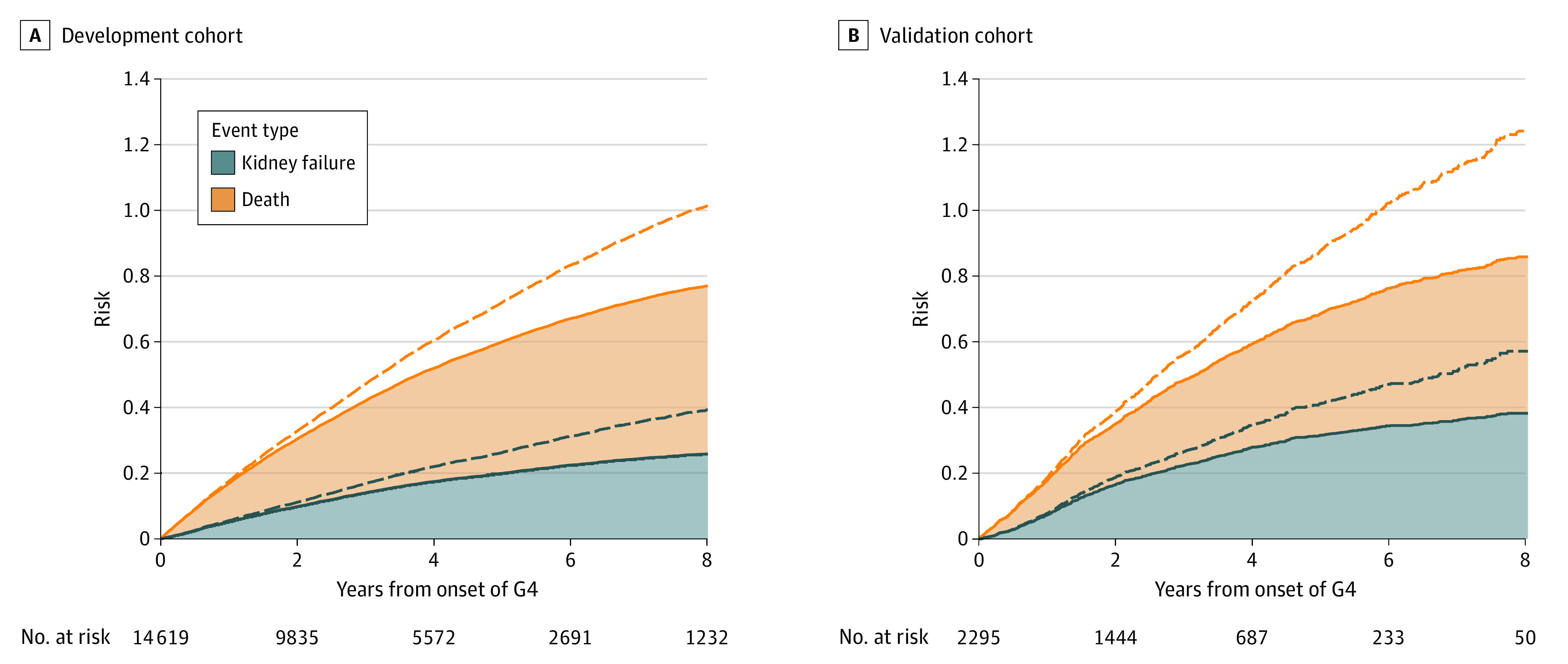
Cumulative Incidence Functions vs Kaplan-Meier Failure Functions Dashed lines indicate Kaplan-Meier estimator functions; solid lines, cumulative incidence functions; G4, severe CKD. All curves are stacked. The sum of the 2 Kaplan-Meier failure functions corresponding to the 2 competing events (kidney failure and death) is more than 1 before year 8 in the development cohort (A) and year 6 in the validation cohort (B).

Figure 1 shows the crude risks in the 2 cohorts estimated by the naïve Kaplan-Meier method and the competing risk method. The naive Kaplan-Meier method results in risk overestimation, which begins to be observed before year 2. The sum of the 2 Kaplan-Meier failure functions corresponding to the 2 competing events (kidney failure and death) was more than 1 before year 8 in the development cohort and year 6 in the validation cohort (which is impossible because probabilities or risks range between 0 and 1).

### Model Development

The final models are summarized in eTable 4 in the Supplement. The association between age and kidney failure was nonlinear, and the proportional assumption was satisfied for the hazard and subdistribution hazard scales by introducing a quadratic term for age. We found significant interactions between age (and its squared term) and cardiovascular disease and between log-albuminuria and cardiovascular disease.

### Model Prediction Performance in Development and Validation Cohorts

#### Calibration

**Figure 2.**
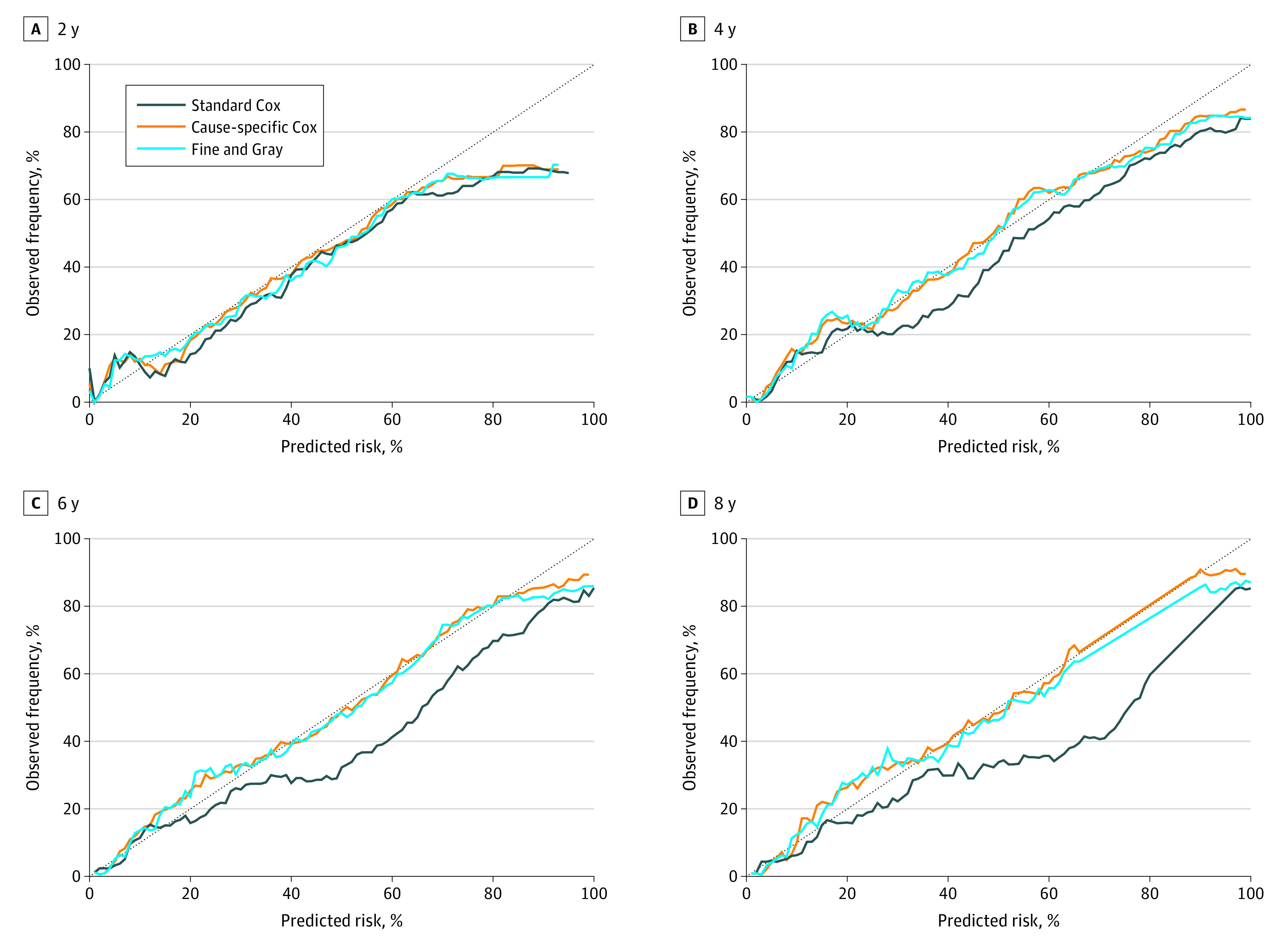
Calibration Plots at 2, 4, 6, and 8 Years in the Validation Cohort Calibration plots summarize the graphical agreement between observed and predicted risks at years 1 through 8. In an ideal model, pairs of the observed and predicted risks lie on a 45-degree angle line. Curves falling under the 45-degree angle line indicate that predicted risks overestimate (are higher than) observed risks. Corresponding plots at years 1 through 8 for the development cohort and for years 1, 3, 5, and 7 for the validation cohort are provided in the eFigures 2-4 in the Supplement.

Calibration of the 3 models was similar in short-term predictions (years 1 to 2). Beyond 2 years, the risks predicted by the standard Cox regression exceeded observed risks, while those from the competing risks models were closer to the 45° angle line for almost the entire risk range (Figure 2; eFigure 2 to 4 in the Supplement). At 4 years, for example, risks predicted from standard Cox were 40% for people whose observed risks were less than 30% (Figure 2).

#### Reclassification

**Figure 3.**
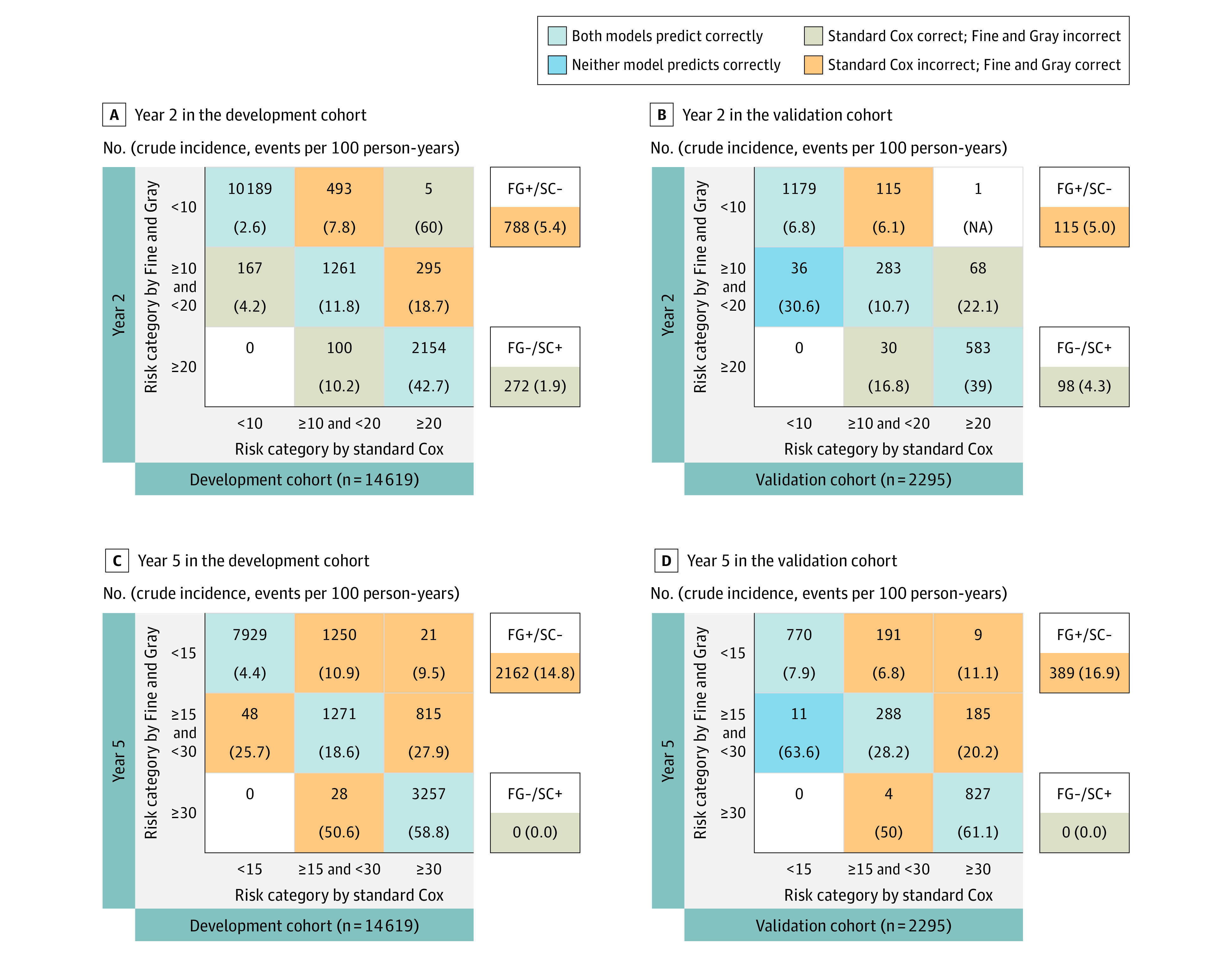
Predicted vs Observed Risk of Kidney Failure at Years 2 and 5 in the Development and Validation Cohorts Risk (%) was predicted for each member of the development (A, C) and validation (B, D) cohort according to the Fine and Gray subdistribution hazard model and the standard Cox model at years 2 and 5 from study entry. People were then assigned to each cell of a 3 × 3 table corresponding to the combination of the model predictions. Each cell of the 3 × 3 table includes the number of people (top) and their actual observed risk (crude cumulative incidence function) at 2 or 5 years (bottom, bold). FG-/SC+, total No. (%) of people incorrectly classified by Fine-Gray model and correctly classified by standard Cox regression with respect to the actual observed risk; FG+/SC-, total No. (%) of people correctly classified by the Fine-Gray model and incorrectly classified by standard Cox regression with respect to the actual observed risk; NA indicates not available.

At 2 and 5 years, 788 people (5.4%) and 2162 people (14.8%) were correctly reclassified into lower- or higher-risk categories by the Fine and Gray model and incorrectly reclassified by standard Cox regression (the opposite was observed in 272 people [1.9%] and 0 people, respectively) in the development cohort (Figure 3). For the comparison between the cause-specific Cox regression and the standard Cox regression, the corresponding figures were 569 people (3.9%) and 2025 people (13.9%) (the opposite was observed 0 people in either cohort) (eFigure 5 in the Supplement). In the validation cohort, 115 (5.0%) and 389 (16.9%) of people at 2 and 5 years, respectively, were correctly reclassified into lower- or higher-risk categories by the Fine and Gray model and incorrectly reclassified by standard Cox regression (the opposite was observed in 98 people [4.3%] and 0 people, respectively). For the comparison between cause-specific Cox regression and standard Cox regression, the corresponding figures were 135 people (5.9%) and 393 people (17.1%) (the opposite was observed in 0 individuals in both cohorts).

#### Brier Score and C Index

**Figure 4.**
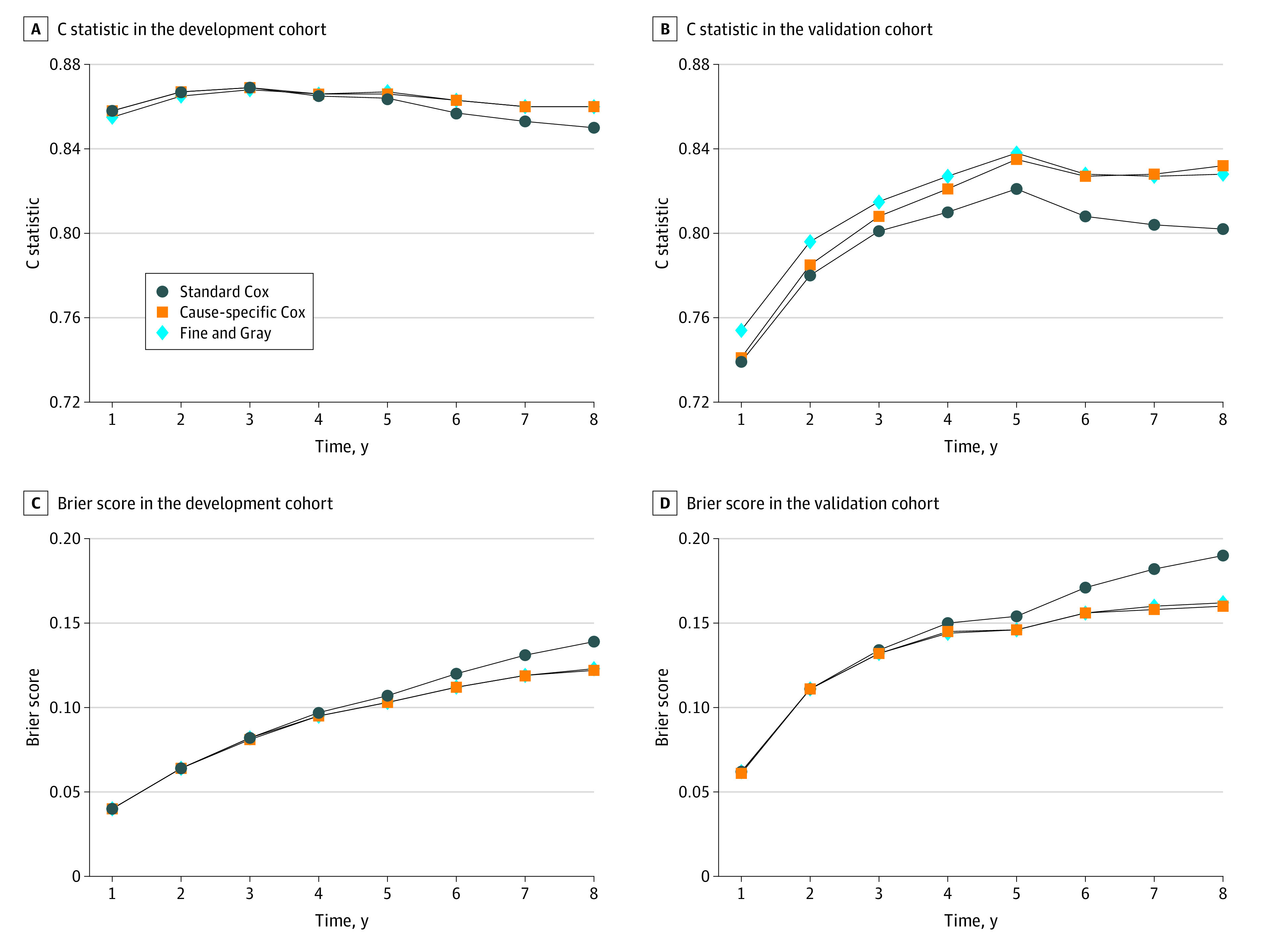
C Statistics and Brier Score in the Development and Validation Cohort The C statistic in the development cohort (A) and validation cohort (B) assesses the ability of separating people with kidney failure from those without kidney failure, ranging from 0.5 (no prediction ability beyond chance) to 1 (perfect discrimination). Smaller Brier scores in the development cohort (C) and validation cohort (D) indicate better performance in terms of both discrimination and calibration.

The time-dependent Brier scores of the 3 models started to separate at 4 to 5 years in the development cohort and at 3 to 4 years in the validation cohort (Figure 4; eTable 5 in the Supplement). The time-dependent C index of the 3 models ranged between 0.84 and 0.87 in the development cohort and between 0.74 and 0.84 in the validation cohort (Figure 4; eTable 5 in the Supplement). The curves of the c index started to separate at 4 to 5 years in the development cohort and at 2 years in the validation cohort (0.85 vs 0.86 and 0.78 vs 0.8, respectively).

### Subgroup and Sensitivity Analysis

Differences in predictions between standard Cox regression and competing risks models were reduced in people at lower risk of death and vice versa (eFigure 6-11 in the Supplement). At 5 years, for example, in people aged 65 years or older, predicted risks from standard Cox were 50% where observed risks were less than 30%. Similar mis-calibration was observed at 5 years in people with albuminuria greater than 30 mg/mmol, diabetes or cardiovascular disease. These differences varied in magnitude at 2 years depending on the thresholds for risk categories used for reclassification but were consistent in magnitude at 5 years across different thresholds (eFigure 12-15, eTable 6 and 7 in the Supplement). For example, at 5 years 17% of people aged 65 years or older were correctly reclassified into lower- or higher-risk categories by competing risk models and incorrectly reclassified by standard Cox regression (vs 5% of people younger than 65 years), and the opposite was observed in 0 people. Reclassification improvement was even more pronounced in people with albuminuria greater than 30 mg/mmol, diabetes or cardiovascular disease (18% to 24%). We obtained similar results in other sensitivity analyses (eFigure 16-18 in the Supplement).

## Discussion

We developed and validated 2 models to predict kidney failure in adults with G4-CKD accounting for the competing risk of death. The risk of kidney failure varies in adults with severe CKD depending on age, sex, index eGFR, degree of albuminuria,^3,4^ and comorbidities.^5,31^ The 2 competing risk models we built with these predictors demonstrated excellent accuracy (defined as well-calibrated models with C index 0.8-0.9) for the entire risk range and prediction time. The standard Cox regression, which does not account for the competing risk of death, provided similar performance during the first 2 years after onset of G4-CKD, but differences began to emerge thereafter, especially among people older than 65 years or with more comorbidities. The traditional approach overestimates the risk of kidney failure for people with a higher risk of death. We built these models using comorbidity and laboratory data as in previous studies.^3,4^ If confirmed in other settings, our findings suggest that existing clinical risk calculators could be updated to use similar methods and perhaps improve the accuracy of predictions about the risk of kidney failure.

Using standard Cox regression to predict kidney failure results in risk overestimation because standard Cox regression treats death as a censoring event. Censoring is assumed to leave the risk of the event of interest unaltered, but the risk of kidney failure becomes 0 after death. However, the impact of censoring for death in an analysis of the risk of kidney failure may be minimal if the competing risk is infrequent.^32^ With longer prediction times or in the presence of a high risk of death relative to the risk of kidney failure, censoring for death could result in kidney failure risk predictions that are increasingly biased upward. This is especially true among people with severe CKD, many of whom are older and have multiple comorbidities. These limitations should be acknowledged if standard Cox regression will be used, for example, because of computational issues with competing risks modeling or because the calculator based on standard Cox regression has already been implemented in the decision support system to guide nephrology referral or inform advanced care planning.^2^

Our findings are consistent with previous studies that showed similar performance of methods that do and do not account for competing risks in short-term predictions and in people who have higher risk of kidney failure than death.^3,4^ Indeed, small differences between these methods may be explained by the inclusion of adults with mild to severe CKD, younger and more selected populations (patients referred to a nephrologist),^6^ and shorter follow-up times (2 to 4 years) in those studies. Our study is novel, as it compares standard Cox regression with competing risks models over a longer follow-up time and using recommended measures of model performance.^8^ We have considered the limitations of Cox models in the competing risks setting by emphasizing the role of calibration^23^ and using adapted reclassification methodology.^24^ Considering that many adults with severe CKD are 75 years or older and have a higher risk of death than kidney failure, our findings suggest that predictions based on a competing risks model may be warranted to minimize the risk of unnecessary interventions or overtreatment.^5^

We used population-based data from a geographically defined area served by a universal health care system, a relatively large sample size, and a long follow-up to develop and validate competing risks models that demonstrated accurate predictions. The excellent external validation results support the use of these equations in diverse populations, including relatively younger and healthier populations in whom the competing risk of death may be lower than in the population we used to develop these models. These models are based on information that is routinely collected in clinical practice, which can be integrated into electronic support systems for clinical management and future decision aids. The use of population-based data suggests that our findings are broadly generalizable to people with severe CKD (rather than the select population who have been referred to nephrologists)^6^ at least in those who are White and reside in North America and Europe. We used rigorous methods, as in our previous studies,^2^ for ascertaining the presence or absence of comorbidity, defining eligibility criteria to align sample characteristics to those of the target population with severe CKD, and maximizing the inclusion of incident patients with severe CKD. Furthermore, we extended our definition of kidney failure to include kidney failure with and without kidney replacement therapy. This is important considering that the decision to initiate dialysis or perform kidney transplantation is partially subjective and that older patients may opt for conservative management of kidney failure.

### Limitations

Our study has limitations, including the use of routinely collected data from people who accessed medical services and the use of data from 2 Canadian provinces with a very low prevalence of people who self-identify as Black. Our study only included people with G4-CKD and had albuminuria information available, which may have contributed to the overrepresentation of patients with diabetes in our cohorts. Although our findings will require confirmation in other settings, we do not believe that these limitations pose a threat to the validity of our results or interpretations.

## Conclusions

These findings suggest that accounting for the competing risk of death when making predictions about kidney failure among people with G4-CKD becomes increasingly important with longer follow-up time, older age, and the presence of more comorbidity. If these findings are confirmed in other settings, existing clinical risk calculators should be updated to use similar methods and may improve predictions about the risk of kidney failure.

## Notes

Supplement. **eTable 1.** Coding Definitions for Baseline Characteristics **eTable 2.** Coding Definitions for Identifying Dialysis or Transplantation **eTable 3.** Baseline Characteristics by Cohort Entry on or Before vs After the Median Index Date (Development Cohort) **eTable 4.** Model Coefficients **eTable 5.** Difference in C Statistics and Brier Scores **eTable 6.** Kidney Failure Risk Reclassification at Year 2 Considering Different Risk Cutoffs: Subgroup Analyses (Development Cohort) **eTable 7.** Kidney Failure Risk Reclassification at Year 5 Considering Different Risk Cutoffs: Subgroup Analyses (Development Cohort) **eFigure 1.** Derivation of Development and Validation Cohorts **eFigure 2.** Calibration Plots at 1, 3, 5, and 7 Years in the Development Cohort **eFigure 3.** Calibration Plots at 2, 4, 6, and 8 Years in the Development Cohort **eFigure 4.** Calibration Plots at 1, 3, 5, and 7 Years in the Validation Cohort **eFigure 5.** Reclassification Table With Cutoffs at 2 Years of 10% and 20% and at 5 Years of 15% and 30% (Cause-Specific Cox) **eFigure 6.** Calibration Plots at 2 and 5 Years by Age (Development Cohort)
**eFigure 7.** Calibration Plots at 2 and 5 Years by Sex (Development Cohort) **eFigure 8. **Calibration Plots at 2 and 5 Years by Albumin-Creatinine Ratio (ACR; Development Cohort) **eFigure 9.** Calibration Plots at 2 and 5 Years by Median Estimated Glomerular Filtration Rate (Development Cohort) **eFigure 10.** Calibration Plots at 2 and 5 Years by Diabetes (Development Cohort) **eFigure 11.** Calibration Plots at 2 and 5 Years by Cardiovascular Disease (Development Cohort) **eFigure 12.** Reclassification Table With Cutoffs at 2 Years of 5% and 15% and at 5 Years of 10% and 20% (Fine and Gray) **eFigure 13.** Reclassification Table With Cutoffs at 2 Years of 5% and 15% and at 5 Years 10% and 20% (Cause-Specific Cox) **eFigure 14.** Reclassification Table With Cutoffs at 2 Years of 10% and 40% and at 5 Years of 5% and 50% (Fine and Gray) **eFigure 15.** Reclassification Table With Cutoffs at 2 Years of 10% and 40% and at 5 Years of 5% and 50% (Cause-Specific Cox) **eFigure 16.** Calibration Plots at 2 and 5 Years by Cohort Entry on or Before vs After the Median Index Date (Development Cohort) **eFigure 17.** Calibration Plots at 1, 3, 5, and 7 Years (Sensitivity Analysis) **eFigure 18.** Calibration Plots at 2, 4, 6, and 8 Years (Sensitivity Analysis) **eReferences.**
