# Supplementary material for: Accounting for the Competing Risk of Death to Predict Kidney Failure in Adults With Stage 4 Chronic Kidney Disease
Source: JAMA Netw Open. 2021 May 4;4(5):e219225. doi: 10.1001/jamanetworkopen.2021.9225 (PMC8097501; doi:10.1001/jamanetworkopen.2021.9225)
Supplement: Supplement. — eTable 1. Coding Definitions for Baseline Characteristics eTable 2. Coding Definitions for Identifying Dialysis or Transplantation eTable 3. Baseline Characteristics by Cohort Entry on or Before vs After the Median Index Date (Development Cohort) eTable 4. Model Coefficients eTable 5. Difference in C Statistics and Brier Scores eTable 6. Kidney Failure Risk Reclassification at Year 2 Considering Different Risk Cutoffs: Subgroup Analyses (Development Cohort) eTable 7. Kidney Failure Risk Reclassification at Year 5 Considering Different Risk Cutoffs: Subgroup Analyses (Development Cohort) eFigure 1. Derivation of Development and Validation Cohorts eFigure 2. Calibration Plots at 1, 3, 5, and 7 Years in the Development Cohort eFigure 3. Calibration Plots at 2, 4, 6, and 8 Years in the Development Cohort eFigure 4. Calibration Plots at 1, 3, 5, and 7 Years in the Validation Cohort eFigure 5. Reclassification Table With Cutoffs at 2 Years of 10% and 20% and at 5 Years of 15% and 30% (Cause-Specific Cox) eFigure 6. Calibration Plots at 2 and 5 Years by Age (Development Cohort) eFigure 7. Calibration Plots at 2 and 5 Years by Sex (Development Cohort) eFigure 8. Calibration Plots at 2 and 5 Years by Albumin-Creatinine Ratio (ACR; Development Cohort) eFigure 9. Calibration Plots at 2 and 5 Years by Median Estimated Glomerular Filtration Rate (Development Cohort) eFigure 10. Calibration Plots at 2 and 5 Years by Diabetes (Development Cohort) eFigure 11. Calibration Plots at 2 and 5 Years by Cardiovascular Disease (Development Cohort) eFigure 12. Reclassification Table With Cutoffs at 2 Years of 5% and 15% and at 5 Years of 10% and 20% (Fine and Gray) eFigure 13. Reclassification Table With Cutoffs at 2 Years of 5% and 15% and at 5 Years 10% and 20% (Cause-Specific Cox) eFigure 14. Reclassification Table With Cutoffs at 2 Years of 10% and 40% and at 5 Years of 5% and 50% (Fine and Gray) eFigure 15. Reclassification Table With Cutoffs at 2 Years of 10% and 40% and at 5 Years of [file jamanetwopen-e219225-s001.pdf]

Supplemental Online Content

Al-Wahsh H, Tangri N, Quinn R, et al. Accounting for the competing risk of death to predict kidney failure in adults with stage 4 chronic kidney disease. *JAMA Netw Open*. 2021;4(5):e219225. doi:10.1001/jamanetworkopen.2021.9225

eTable 1. Coding Definitions for Baseline Characteristics
eTable 2. Coding Definitions for Identifying Dialysis or Transplantation
eTable 3. Baseline Characteristics by Cohort Entry on or Before vs After the Median Index Date (Development Cohort)
eTable 4. Model Coefficients
eTable 5. Difference in C Statistics and Brier Scores
eTable 6. Kidney Failure Risk Reclassification at Year 2 Considering Different Risk Cutoffs: Subgroup Analyses (Development Cohort)
eTable 7. Kidney Failure Risk Reclassification at Year 5 Considering Different Risk Cutoffs: Subgroup Analyses (Development Cohort)
eFigure 1. Derivation of Development and Validation Cohorts
eFigure 2. Calibration Plots at 1, 3, 5, and 7 Years in the Development Cohort
eFigure 3. Calibration Plots at 2, 4, 6, and 8 Years in the Development Cohort
eFigure 4. Calibration Plots at 1, 3, 5, and 7 Years in the Validation Cohort
eFigure 5. Reclassification Table With Cutoffs at 2 Years of 10% and 20% and at 5 Years of 15% and 30% (Cause-Specific Cox)
eFigure 6. Calibration Plots at 2 and 5 Years by Age (Development Cohort)
eFigure 7. Calibration Plots at 2 and 5 Years by Sex (Development Cohort)
eFigure 8. Calibration Plots at 2 and 5 Years by Albumin-Creatinine Ratio (ACR; Development Cohort)
eFigure 9. Calibration Plots at 2 and 5 Years by Median Estimated Glomerular Filtration Rate(Development Cohort)
eFigure 10. Calibration Plots at 2 and 5 Years by Diabetes (Development Cohort)
eFigure 11. Calibration Plots at 2 and 5 Years by Cardiovascular Disease (Development Cohort)
eFigure 12. Reclassification Table With Cutoffs at 2 Years of 5% and 15% and at 5 Years of 10% and 20% (Fine and Gray)
eFigure 13. Reclassification Table With Cutoffs at 2 Years of 5% and 15% and at 5 Years 10% and 20% (Cause-Specific Cox)
eFigure 14. Reclassification Table With Cutoffs at 2 Years of 10% and 40% and at 5 Years of 5% and 50% (Fine and Gray)
eFigure 15. Reclassification Table With Cutoffs at 2 Years of 10% and 40% and at 5 Years of 5% and 50% (Cause-Specific Cox)
eFigure 16. Calibration Plots at 2 and 5 Years by Cohort Entry on or Before vs After the Median Index Date (Development Cohort)
eFigure 17. Calibration Plots at 1, 3, 5, and 7 Years (Sensitivity Analysis)
eFigure 18. Calibration Plots at 2, 4, 6, and 8 Years (Sensitivity Analysis)
eReferences.

This supplemental material has been provided by the authors to give readers additional information about their work.

eTable 1. Coding Definitions for Baseline Characteristics[1]

Variable	Codes	Validation
Diabetes mellitus	1 hospitalization or 2 claims in 2 years or less:<br>ICD-9-CM: 250<br>ICD-10: E10-E14	ICD-9-CM: Sn 79%, PPV 95%<br>ICD-10: Sn 68%, PPV 93%
Myocardial infarction	1 hospitalization:<br>ICD-9-CM: 410<br>ICD-10: I21, I22	ICD-9-CM: Sn 89%, PPV 89%
Heart failure	1 hospitalization or 2 claims in 2 years or less:<br>ICD-9-CM: 398.91, 402.01, 402.11, 402.91, 404.01, 404.03, 404.11, 404.13, 404.91, 404.93, 425.4-425.9, 428<br>ICD-10: I09.9, I25.5, I42.0, I42.5-I42.9, I43, I50	ICD-9-CM: Sn 72%, PPV 91%<br>ICD-10: Sn 69%, PPV 90%
Stroke/Transient ischemia attack	1 most responsible or post-admittance hospitalization or 1 claim or 1 most emergency department ACCS:<br>ICD-9-CM: 362.34, 430, 431, 433, 434, 435, 436<br>ICD-10: G45, H34.0, I60, I61, I63, I64	ICD-9-CM: PPV 90%<br>ICD-10: PPV 92%
Peripheral vascular disease	1 hospitalization or 1 claim or 1 ACCS:<br>ICD-9-CM: 440.2<br>ICD-10: I70.2	ICD-9-CM: Sn 77%, PPV 94%

Abbreviations: ACCS: Ambulatory Care Classification System; ICD-9-CM, International Classification of Diseases, Ninth Revision, Clinical Modification; ICD-10, International Statistical Classification of Diseases, Tenth Revision; PPV, positive predictive value; Sn, sensitivity.

© 2021 Al-Wahsh H et al. *JAMA Network Open.*

eTable 2. Coding Definitions for Identifying Dialysis or Transplantation

Physician claims: Canadian Classification of Diagnostic, Therapeutic, and Surgical Procedures codes

Codes	Code description
For dialysis	
13.99A	Hemodialysis treatment, unstable patient
13.99B	Hemodialysis treatment, stable patient
13.99C	Assessment and management of an unstable patient with acute/chronic renal
13.99D	Assessment and management of a stable patient with chronic renal failure
13.99O	Management of dialysis patients on home dialysis or receiving treatment in a
13.99OA	Management of patient on hemodialysis or peritoneal dialysis (per week)
13.99AB	Dialysis therapy, any modality, in the intensive care unit
For transplantation	
67.5	Transplant of kidney
67.59	Other kidney transplantation
67.59A	Renal transplantation (homo, hetero, auto)

Hospitalizations: Canadian Classification of Health Intervention codes

Codes	Code description
For transplantation	
1.PC.85.^^	Transplant, kidney
1.PC.85.LA-XX-J	Using living donor (allogenic or syngeneic) kidney
1.PC.85.LA-XX-K	Using deceased donor kidney
1.OK.85.XU-XX-K	Transplant, pancreas with duodenum and kidney
1.OK.85.XV-XX-K	Transplant, pancreas with duodenum and kidney

© 2021 Al-Wahsh H et al. *JAMA Network Open.*

eTable 3. Baseline Characteristics on or Before and After the Media Index Date (Development Cohort)

Characteristics	Before or on 19-Oct-2009 (N=7,313)	After 19-Oct-2009 (N = 7,306)
Age, in years, mean (SD)	73.7 (12.4)	74.5 (13.2)
Men, No. (%)	3,484 (47.6)	3,586 (49.1)
eGFR, in ml/min/1.73 m^2, median (IQR)	27.3 (24.6 – 28.9)	27.8 (25.6 – 29.1)
ACR, in mg/mmol, median (IQR)	8.0 (1.8 – 63.8)	7.1 (1.5 – 50.1)
Diabetes mellitus, No. (%)	4,947 (67.6)	4,939 (67.6)
Cardiovascular disease, No. (%)	4,075 (55.7)	4,210 (57.6)
Myocardial infraction, No. (%)	882 (12.1)	909 (12.4)
Congestive heart failure, No. (%)	2,888 (39.5)	2,847 (39.0)
Stroke or TIA, No. (%)	1,684 (23.0)	1,910 (26.1)
Peripheral vascular disease, No. (%)	580 (7.9)	637 (8.7)
Follow-up time, in years, median (IQR)	3.3 (1.5 – 6.6)	3.2 (1.5 – 4.5)
Competing events		
Kidney failure, No. (%)	1,928 (26.4)	1,337 (18.3)
Incidence per 100 person-years (95% CI)	6.4 (6.1 – 6.7)	5.9 (5.5 – 6.2)
Death without kidney failure, No. (%)	3,638 (49.7)	2,890 (39.6)
Incidence per 100 person-years (95% CI)	12.0 (11.6 – 12.4)	12.7 (12.2 – 13.1)

Abbreviations: ACR, albumin-to-creatinine ratio; eGFR, estimated glomerular-filtration rate; IQR, interquartile range; SD, standard deviation; TIA, transient ischemic attack; 95% CI, 95% confidence interval.

© 2021 Al-Wahsh H et al. *JAMA Network Open.*

eTable 4. Model Coefficients

	Cause-specific Cox models		*Fine and Gray model*
Coefficient	***HR of kidney failure (95% CI)***	***HR of death (95% CI)***	***SHR of kidney failure (95% CI)***
Age (10 years)	0.71 (0.66-0.75)	1.99 (1.90-2.09)	0.58 (0.55-0.62)
Age^2 (squared term)	0.98 (0.96-0.99)	1.06 (1.03-1.08)	0.94 (0.93-0.96)
Male sex (vs female)	1.27 (1.16-1.39)	1.26 (1.19-1.33)	1.21 (1.11-1.32)
Log ACR (log mg/mmol)	1.47 (1.41-1.53)	1.09 (1.07-1.12)	1.41 (1.36-1.47)
eGFR (5 mL/min/1.73 m^2)	0.56 (0.50-0.62)	0.76 (0.70-0.82)	0.61 (0.54-0.68)
CV (vs no CV)	1.12 (0.92-1.37)	2.27 (2.06-2.49)	0.88 (0.71-1.08)
Diabetes (vs no diabetes)	0.90 (0.74-1.10)	1.20 (1.14-1.27)	0.93 (0.76-1.13)
Male sex * eGFR	1.09 (1.00-1.20)	1.10 (1.02-1.19)	1.06 (0.96-1.17)
CV * eGFR	0.93 (0.85-1.02)	1.07 (0.98-1.17)	0.91 (0.82-1.00)
Diabetes * eGFR	0.92 (0.82-1.02)	-	0.94 (0.84-1.05)
Diabetes * Log ACR	1.06 (1.02-1.11)	-	1.04 (1.00-1.09)
CV * Age	0.79 (0.72-0.87)	0.87 (0.82-0.92)	0.73 (0.66-0.80)
CV * Age^2	0.95 (0.92-0.98)	0.99 (0.95-1.02)	0.94 (0.91-0.97)
CV * Log ACR	0.94 (0.90-0.97)	0.98 (0.95-1.01)	0.95 (0.91-0.99)
CV * Diabetes	1.15 (0.96-1.38)	-	1.06 (0.88-1.27)

Abbreviation: HR, hazard ratio; SHR, sub-distribution hazard ratio; CI, confidence interval. ACR, albumin-to-creatinine ratio; CV, cardiovascular disease; eGFR, estimated glomerular filtration rate.

© 2021 Al-Wahsh H et al. *JAMA Network Open.*

eTable 5. Difference in C statistics and Brier Scores

			Development cohort					Validation cohort				
		Year	*Diff.*	*95% L*	*95% U*	*P value*	*Interpretation*	*Diff.*	*95% L*	*95% U*	*P value*	*Interpretation*
C-stratistics	*Cause-specific Cox vs Standard Cox*	1	0.00	0.00	0.00	0.90	Non-significant	0.30	0.10	0.40	<0.001	Favor Cause-specific Cox
		2	0.00	-0.10	0.10	0.80	Non-significant	0.50	0.30	0.70	<0.001	Favor Cause-specific Cox
		3	0.10	0.00	0.20	0.30	Non-significant	0.70	0.50	1.00	<0.001	Favor Cause-specific Cox
		4	0.10	0.00	0.30	0.03	Favor Cause-specific Cox	1.10	0.80	1.40	<0.001	Favor Cause-specific Cox
		5	0.30	0.10	0.40	<0.001	Favor Cause-specific Cox	1.40	0.90	1.80	<0.001	Favor Cause-specific Cox
		6	0.60	0.40	0.80	<0.001	Favor Cause-specific Cox	1.90	1.30	2.40	<0.001	Favor Cause-specific Cox
		7	0.70	0..5	1.00	<0.001	Favor Cause-specific Cox	2.30	1.80	3.00	<0.001	Favor Cause-specific Cox
		8	1.00	0.70	1.20	<0.001	Favor Cause-specific Cox	2.60	2.20	3.80	<0.001	Favor Cause-specific Cox
	Fine and Gray vs Standard Cox	1	-0.30	-0.60	0.00	0.04	Favor Standard Cox	1.50	0.70	2.40	<0.001	Favor Fine and Gray
		2	-0.20	-0.40	0.00	0.07	Non-significant	1.60	0.90	2.20	<0.001	Favor Fine and Gray
		3	-0.10	-0.30	0.10	0.40	Non-significant	1.50	0.90	2.10	<0.001	Favor Fine and Gray
		4	0.10	-0.10	0.30	0.40	Non-significant	1.70	1.10	2.20	<0.001	Favor Fine and Gray
		5	0.30	0.10	0.50	0.004	Favor Fine and Gray	1.70	1.10	2.20	<0.001	Favor Fine and Gray
		6	0.60	0.40	0.80	<0.001	Favor Fine and Gray	2.00	1.40	2.60	<0.001	Favor Fine and Gray
		7	0.80	0.60	1.00	<0.001	Favor Fine and Gray	2.30	1.70	2.90	<0.001	Favor Fine and Gray
		8	1.00	0.80	1.20	<0.001	Favor Fine and Gray	2.60	2.00	3.20	<0.001	Favor Fine and Gray
Brier score	*Cause-specific Cox vs Standard Cox*	1	0.00	0.00	0.00	0.38	Non-significant	0.00	0.00	0.00	0.008	Favor Cause-specific Cox
		2	0.00	0.00	0.00	0.83	Non-significant	-0.10	-0.10	0.00	0.08	Non-significant
		3	-0.10	-0.10	0.00	0.06	Non-significant	-0.20	-0.40	-0.10	0.003	Favor Cause-specific Cox
		4	-0.20	-0.30	-0.10	<0.001	Favor Cause-specific Cox	-0.50	-0.70	-0.20	<0.001	Favor Cause-specific Cox
		5	-0.40	-0.50	-0.30	<0.001	Favor Cause-specific Cox	-0.80	-1.10	-0.40	<0.001	Favor Cause-specific Cox
		6	-0.80	-1.00	-0.60	<0.001	Favor Cause-specific Cox	-1.50	-1.90	-1.00	<0.001	Favor Cause-specific Cox
		7	-1.20	-1.50	-1.00	<0.001	Favor Cause-specific Cox	-2.40	-3.00	-1.80	<0.001	Favor Cause-specific Cox
		8	-1.70	-2.00	-1.30	<0.001	Favor Cause-specific Cox	-3.10	-3.50	-2.30	<0.001	Favor Cause-specific Cox
	Fine and Gray vs Standard Cox	1	0.00	0.00	0.10	0.002	Favor Standard Cox	0.00	-0.10	0.10	0.83	Non-significant
		2	0.10	0.00	0.10	0.02	Favor Standard Cox	-0.10	-0.20	0.10	0.42	Non-significant
		3	0.00	-0.10	0.10	0.54	Non-significant	-0.20	-0.40	0.00	0.02	Favor Fine and Gray
		4	-0.20	-0.30	-0.10	<0.001	Favor Fine and Gray	-0.50	-0.80	-0.30	<0.001	Favor Fine and Gray
		5	-0.40	-0.60	-0.30	<0.001	Favor Fine and Gray	-0.80	-1.10	-0.50	<0.001	Favor Fine and Gray
		6	-0.80	-1.00	-0.60	<0.001	Favor Fine and Gray	-1.40	-1.90	-1.00	<0.001	Favor Fine and Gray
		7	-1.20	-1.40	-1.00	<0.001	Favor Fine and Gray	-2.20	-2.80	-1.70	<0.001	Favor Fine and Gray
		8	-1.60	-1.90	-1.30	<0.001	Favor Fine and Gray	-2.80	-3.50	-2.10	<0.001	Favor Fine and Gray

Legend: Diff. indicates the difference in C statistics or Brier score*100; L and U indicates lower and upper limit of the 95% confidence intervals.

© 2021 Al-Wahsh H et al. *JAMA Network Open.*

eTable 6. Kidney Failure Risk Reclassification at Year 2 Considering Different Risk Cutoffs: Subgroup Analyses (Development Cohort)

		Cutoffs = 5% and 15%		Cutoffs = 10% and 40%		Cutoffs = 10% and 20%	
Subgroups	Model	CR+/SC-	CR-/SC+	CR+/SC-	CR-/SC+	CR+/SC-	CR-/SC+
Age >65 years (N=11,564)	CS	494 (4.3)	264 (2.3)	313 (2.7)	53 (0.5)	500 (4.3)	0 (0.0)
	FG	**1,260 (10.9)**	14 (0.1)	497 (4.3)	120 (1.0)	769 (6.6)	5 (0.0)
Age ≤65 years (N=3,055)	CS	0 (0.0)	62 (2.0)	79 (2.6)	0 (0.0)	45 (1.5)	24 (0.8)
	FG	0 (0.0)	269 (8.8)	51 (1.7)	207 (6.8)	18 (0.6)	243 (8.0)
Males (N=7,070)	CS	491 (6.9)	0 (0.0)	200 (2.8)	66 (0.9)	345 (4.8)	0 (0.0)
	FG	694 (9.8)	177 (2.5)	387 (5.5)	122 (1.7)	484 (6.8)	127 (1.8)
Females (N=7,549)	CS	227 (3.0)	102 (1.4)	137 (1.8)	42 (0.6)	224 (3.0)	0 (0.0)
	FG	337 (4.5)	345 (4.6)	199 (2.6)	168 (2.2)	196 (2.6)	202 (2.7)
ACR >30 mg/mmol (N=4,662)	CS	0 (0.0)	395 (8.5)	233 (5.0)	105 (2.3)	448 (9.6)	0 (0.0)
	FG	**716 (15.4)**	61 (1.3)	390 (8.4)	229 (4.9)	664 (14.2)	91 (2.0)
ACR ≤30 mg/mmol (N=9,957)	CS	358 (3.6)	67 (0.1)	107 (1.1)	0 (0.0)	121 (1.2)	0 (0.0)
	FG	458 (4.6)	318 (3.2)	108 (1.1)	147 (1.5)	108 (1.1)	181 (1.8)
Diabetes (N=9,886)	CS	642 (6.5)	0 (0.0)	279 (2.8)	99 (1.0)	482 (4.9)	0 (0.0)
	FG	**1,167 (11.8)**	0 (0.0)	436 (4.4)	261 (2.6)	712 (7.2)	136 (1.4)
No diabetes (N=4,733)	CS	0 (0.0)	178 (3.8)	0 (0.0)	67 (1.4)	0 (0.0)	87 (1.8)
	FG	168 (3.5)	218 (4.6)	0 (0.0)	179 (3.8)	14 (0.3)	146 (3.1)
CV (N=8,285)	CS	599 (7.2)	0 (0.0)	242 (2.9)	75 (0.9)	415 (5.0)	0 (0.0)
	FG	**985 (11.9)**	41 (0.5)	425 (5.1)	117 (1.4)	658 (7.9)	16 (0.2)
No CV (N=6,334)	CS	120 (1.9)	101 (1.6)	95 (1.5)	33 (0.5)	154 (2.4)	0 (0.0)
	FG	198 (3.1)	329 (5.2)	96 (1.5)	238 (3.8)	153 (2.4)	233 (3.7)
eGFR >27.5 ml/min/1.73 m^2 (N =7310)	CS	307 (4.2)	0 (0.0)	139 (1.9)	0 (0.0)	190 (2.6)	0 (0.0)
	FG	421 (5.8)	210 (2.9)	181 (2.5)	121 (1.7)	227 (3.1)	145 (2.0)
eGFR ≤27.5 ml/min/1.73 m^2 (N =7309)	CS	303 (4.1)	210 (2.9)	214 (2.9)	92 (1.3)	379 (5.2)	0 (0.0)
	FG	408 (5.6)	514 (7.0)	335 (4.6)	241 (3.3)	330 (4.5)	306 (4.2)
Index date > 19-Oct-2009 (N =7306)	CS	401 (5.5)	0 (0.0)	209 (2.9)	0 (0.0)	270 (3.7)	0 (0.0)
	FG	542 (7.4)	172 (2.4)	301 (4.1)	114 (1.6)	240 (3.3)	211 (2.9)
Index date ≤ 19-Oct-2009 (N =7313)	CS	419 (5.7)	0 (0.0)	174 (2.4)	62 (0.8)	299 (4.1)	0 (0.0)
	FG	632 (8.6)	207 (2.8)	255 (3.5)	206 (2.8)	421 (5.8)	134 (1.8)

© 2021 Al-Wahsh H et al. *JAMA Network Open.*

Abbreviation: CR, competing risks; SC, standard Cox; CS, cause-specific Cox; FG, Fine and Gray; CR+/SC-, number (%) of people reclassified correctly by the CR model and incorrectly by the SC model; CR-/SC+ number (%) of people reclassified incorrectly by the CR model and correctly by the SC model. CV, cardiovascular disease; ACR, albumin-creatinine ratio. Bold text indicates proportions exceeding 10%.

© 2021 Al-Wahsh H et al. *JAMA Network Open.*

eTable 7. Kidney Failure Risk Reclassification at Year 5 Considering Different Risk Cutoffs: Subgroup Analyses (Development Cohort)

		Cutoffs = 10% and 20%		Cutoffs = 10% and 40%		Cutoffs = 15% and 30%	
Subgroups	Model	CR+/SC-	CR-/SC+	CR+/SC-	CR-/SC+	CR+/SC-	CR-/SC+
Age >65 years (N=11,564)	CS	**2,416 (20.9)**	0 (0.0)	**2,097 (18.1)**	0 (0.0)	**1,886 (16.3)**	0 (0.0)
	FG	**2,561 (22.1)**	23 (0.2)	**2,246 (19.4)**	34 (0.3)	**2,020 (17.5)**	0 (0.0)
Age ≤65 years (N=3,055)	CS	0 (0.0)	114 (3.7)	157 (5.1)	7 (0.2)	139 (4.5)	0 (0.0)
	FG	93 (3.0)	14 (0.5)	142 (4.6)	6 (0.2)	140 (4.6)	0 (0.0)
Males (N=7,070)	CS	**1,472 (20.8)**	0 (0.0)	**1,164 (16.5)**	0 (0.0)	**1,185 (16.8)**	0 (0.0)
	FG	**1,487 (21.0)**	0 (0.0)	**1,203 (17.0)**	0 (0.0)	**1,236 (17.5)**	0 (0.0)
Females (N=7,549)	CS	**1,058 (14.0)**	0 (0.0)	**1,097 (14.5)**	0 (0.0)	**828 (11.0)**	0 (0.0)
	FG	**1,108 (14.7)**	63 (0.8)	**1,171 (15.5)**	52 (0.7)	**916 (12.1)**	0 (0.0)
ACR >30 mg/mmol (N=4,662)	CS	**848 (18.2)**	0 (0.0)	**650 (13.9)**	0 (0.0)	**1,065 (22.8)**	0 (0.0)
	FG	**979 (21.0)**	0 (0.0)	**668 (14.3)**	5 (0.1)	**1,192 (25.6)**	0 (0.0)
ACR ≤30 mg/mmol (N=9,957)	CS	**1,674 (16.8)**	0 (0.0)	**1,611 (16.2)**	0 (0.0)	959 (9.6)	0 (0.0)
	FG	**1,702 (17.1)**	0 (0.0)	**1,754 (17.6)**	0 (0.0)	965 (9.7)	0 (0.0)
Diabetes (N=9,886)	CS	**1,896 (19.2)**	0 (0.0)	**1,617 (16.4)**	0 (0.0)	**1,580 (16.0)**	0 (0.0)
	FG	**2,035 (20.6)**	16 (0.2)	**1,753 (17.7)**	0 (0.0)	**1,721 (17.4)**	0 (0.0)
No diabetes (N=4,733)	CS	227 (4.8)	407 (8.6)	**644 (13.6)**	0 (0.0)	299 (6.3)	134 (2.8)
	FG	217 (4.6)	429 (9.1)	**669 (14.1)**	6 (0.1)	329 (7.0)	111 (2.3)
CV (N=8,285)	CS	**1,827 (22.1)**	0 (0.0)	**1,676 (20.2)**	0 (0.0)	**1,427 (17.2)**	11 (0.1)
	FG	**1,965 (23.7)**	3 (0.0)	**1,874 (22.6)**	0 (0.0)	**1,537 (18.6)**	0 (0.0)
No CV (N=6,334)	CS	**700 (11.1)**	0 (0.0)	585 (9.2)	0 (0.0)	334 (5.3)	252 (4.0)
	FG	**649 (10.2)**	78 (1.2)	496 (7.8)	56 (0.9)	373 (5.9)	250 (3.9)
eGFR >27.5 ml/min/1.73 m^2 (N =7310)	CS	**1,058 (15.5)**	0 (0.0)	**1,093 (15.0)**	0 (0.0)	**804 (11.0)**	0 (0.0)
	FG	**1,165 (15.9)**	0 (0.0)	**1,190 (16.3)**	4 (0.0)	583 (8.0)	296 (4.0)
eGFR ≤27.5 ml/min/1.73 m^2 (N =7309)	CS	**1,466 (20.1)**	0 (0.0)	**1,168 (16.0)**	0 (0.0)	**1,221 (16.7)**	0 (0.0)
	FG	**1,509 (20.6)**	23 (0.3)	**1,230 (16.8)**	0 (0.0)	**1,281 (17.5)**	0 (0.0)
Index date > 19-Oct-2009 (N =7306)	CS	**1,193 (16.3)**	0 (0.0)	**1,146 (15.7)**	0 (0.0)	**973 (13.3)**	0 (0.0)
	FG	**1,223 (16.7)**	36 (0.5)	**1,235 (16.9)**	0 (0.0)	**991 (13.6)**	0 (0.0)
Index date ≤ 19-Oct-2009 (N =7313)	CS	**1,301 (17.8)**	21 (0.3)	**1,115 (15.2)**	0 (0.0)	**1,048 (14.3)**	0 (0.0)
	FG	**1,409 (19.3)**	0 (0.0)	**1,187 (16.2)**	4 (0.0)	694 (9.5)	441 (6.0)

© 2021 Al-Wahsh H et al. *JAMA Network Open.*

Abbreviation: CR, competing risks; SC, standard Cox; CS, cause-specific Cox; FG, Fine and Gray; CR+/SC-, number (%) of people reclassified correctly by the CR model and incorrectly by the SC model; CR-/SC+ number (%) of people reclassified incorrectly by the CR model and correctly by the SC model. CV, cardiovascular disease; ACR, albumin-creatinine ratio. Bold text indicates proportions exceeding 10%.

© 2021 Al-Wahsh H et al. *JAMA Network Open.*

eFigure 1. Derivation of Development and Validation Cohorts

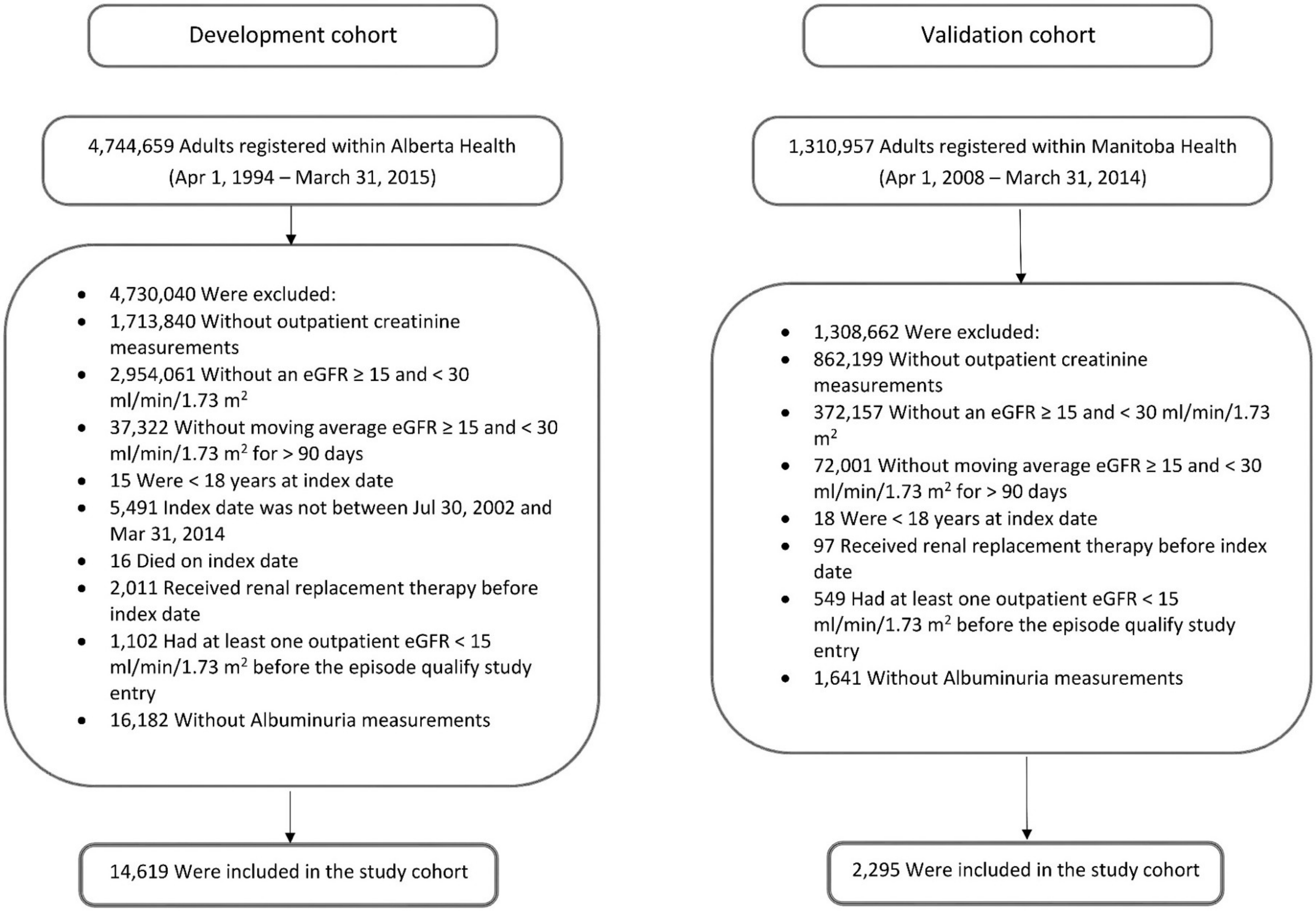

© 2021 Al-Wahsh H et al. *JAMA Network Open.*

eFigure 2. Calibration Plots at 1, 3, 5, and 7 Years (Development Cohort)

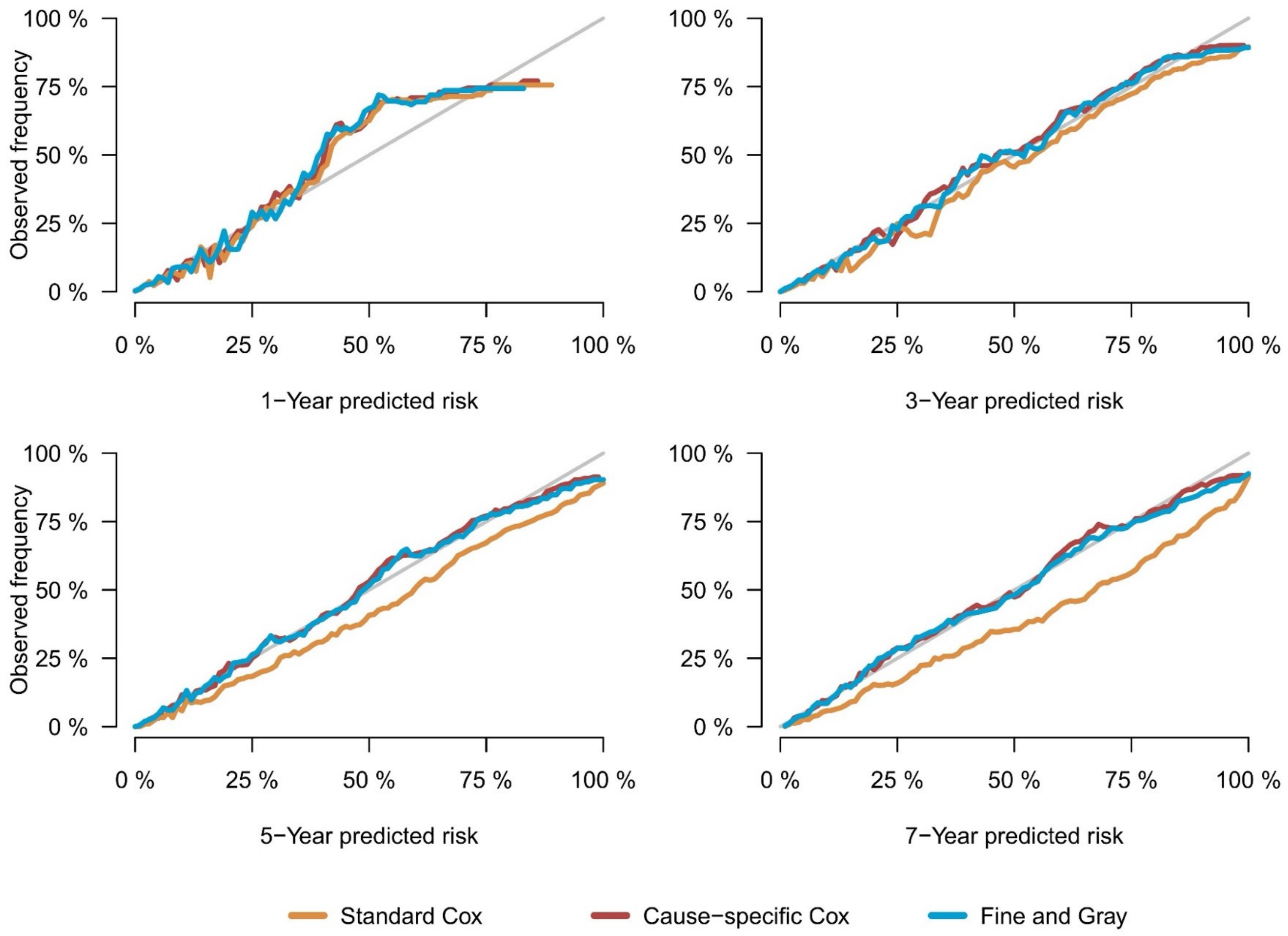

Legend: graphical agreement between observed and predicted risks at years 1-8. In an ideal model, pairs of the observed and predicted risks lie on a45-degree angle line. Curves falling under the 45-degree angle line indicate that predicted risks overestimate (are higher than) observed risks.

© 2021 Al-Wahsh H et al. *JAMA Network Open.*

eFigure 3: Calibration Plots at 2, 4, 6, and 8 Years (Development Cohort)

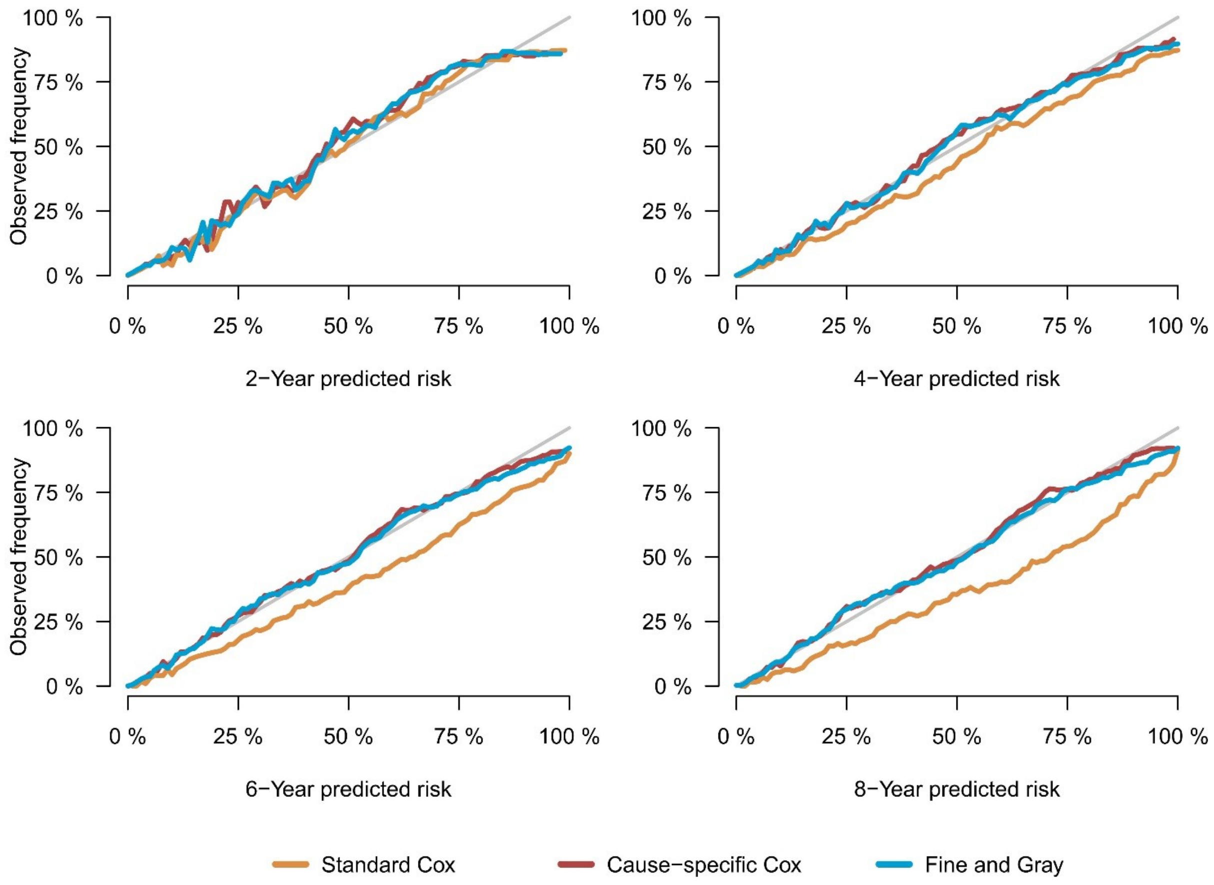

Legend: graphical agreement between observed and predicted risks at years 1-8. In an ideal model, pairs of the observed and predicted risks lie on a 45-degree angle line. Curves falling under the 45-degree angle line indicate that predicted risks overestimate (are higher than) observed risks.

© 2021 Al-Wahsh H et al. *JAMA Network Open.*

eFigure 4: Calibration Plots at 1, 3, 5, and 7 Years (Validation Cohort)

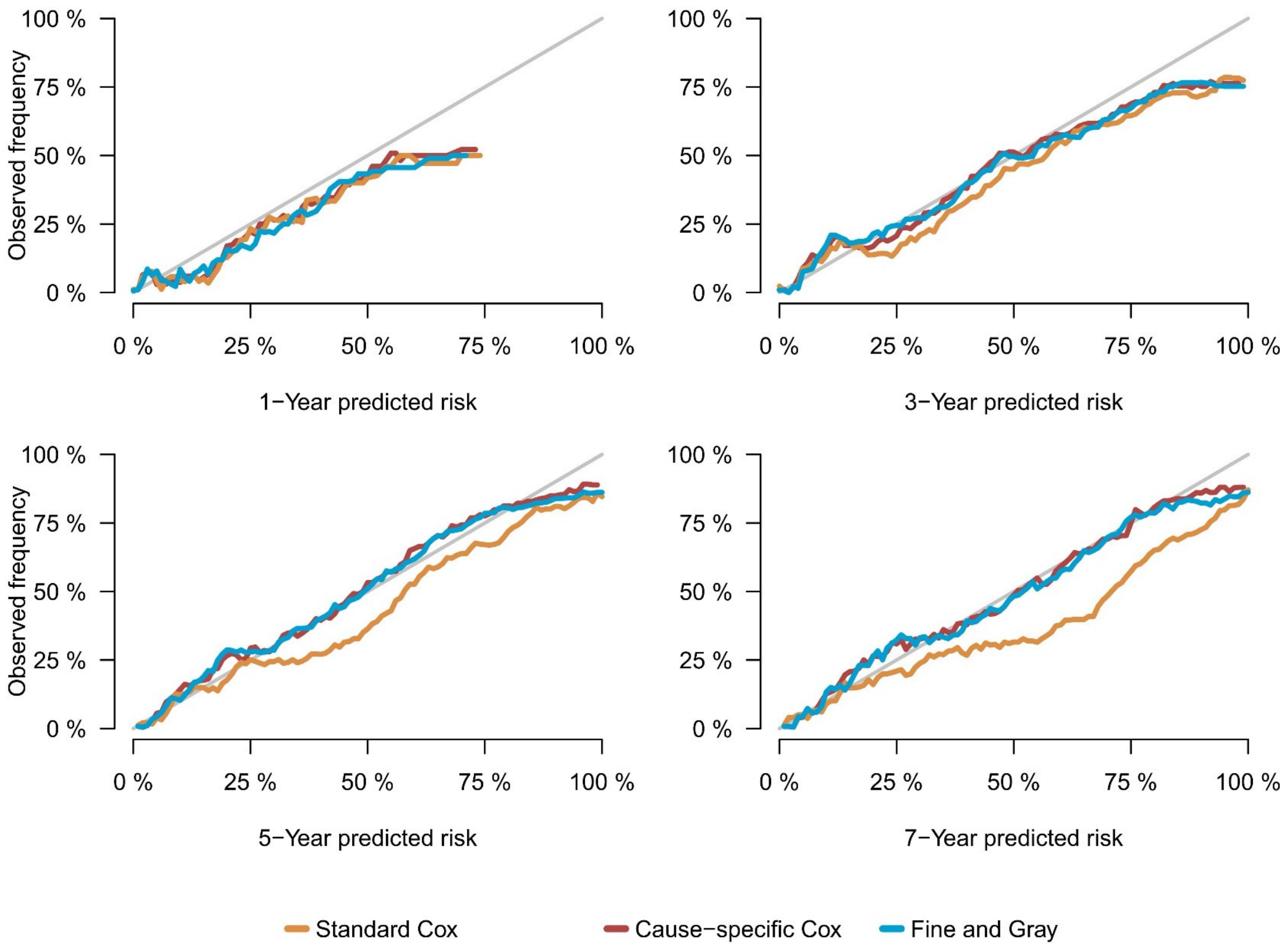

Legend: graphical agreement between observed and predicted risks at years 1-8. In an ideal model, pairs of the observed and predicted risks lie on a 45-degree angle line. Curves falling under the 45-degree angle line indicate that predicted risks overestimate (are higher than) observed risks.

© 2021 Al-Wahsh H et al. *JAMA Network Open.*

eFigure 5. Reclassification Table With Cutoffs at 2 Years of 10% and 20% and at 5 Years of 15% and 30% (Cause-Specific Cox)

Year 2 — Development cohort (N=14,619)

Risk category by cause-specific	Risk category by standard Cox: <10	≥10 & < 20	≥20
<10	10,356<br>**2.6**	337<br>**6.6**	0
≥ 10 & < 20	0	1,472<br>**11.5**	232<br>**14.4**
≥ 20	0	0	2,222<br>**42.4**

CS+/SC-: 569 (3.9)

CS-/SC+: 0 (0.0)

Year 2 — Validation cohort (N=2,295)

Risk category by cause-specific	Risk category by standard Cox: <10	≥10 & < 20	≥20
<10	1,215<br>**7.5**	86<br>**9.3**	0
≥ 10 & < 20	0	342<br>**10.0**	49<br>**14.3**
≥ 20	0	0	603<br>**39.1**

CS+/SC-: 135 (5.9)

CS-/SC+: 0 (0.0)

Year 5 — Development cohort

Risk category by cause-specific	Risk category by standard Cox: <15	≥15 & < 30	≥30
<15	7,977<br>**4.6**	1,212<br>**10.8**	12<br>**8.3**
≥ 15 & < 30	0	1,337<br>**19.2**	801<br>**27.1**
≥ 30	0	0	3,280<br>**58.7**

CS+/SC-: 2,025 (13.9)

CS-/SC+: 0 (0.0)

Year 5 — Validation cohort

Risk category by cause-specific	Risk category by standard Cox: <15	≥15 & < 30	≥30
<15	781<br>**8.7**	195<br>**8.8**	7<br>**0.0**
≥ 15 & < 30	0	288<br>**27.4**	191<br>**21.3**
≥ 30	0	0	833<br>**61**

CS+/SC-: 393 (17.1)

CS-/SC+: 0 (0.0)

Color	Meaning	Color	Meaning
Green	Both models predict correctly	Yellow	Standard Cox correct; cause-specific Cox incorrect
Blue	Neither model predicts correctly	Pink	Standard Cox incorrect; cause-specific Cox correct

Legend: Risk (%) was predicted for each member of the development (left) and validation (right) cohort according to the cause-specific Cox and standard Cox models at years 2 and 5 from study entry. People were then assigned to each cell of a 3X3 table corresponding to the combination of the model predictions. Each cell of the 3X3 table includes the number of people (top) and their actual observed risk (crude cumulative incidence function) at 2 or 5 years (bottom, bold). CS-/SC+, total N and

% of people incorrectly classified by cause-specific Cox and correctly classified by standard Cox regression with respect to the actual observed risk; CS+/SC-, total N and % of people correctly classified by cause-specific Cox and incorrectly classified by standard Cox regression with respect to the actual observed risk.

© 2021 Al-Wahsh H et al. *JAMA Network Open.*

eFigure 6. Calibration Plots at 2 and 5 Years by Age (Development Cohort)

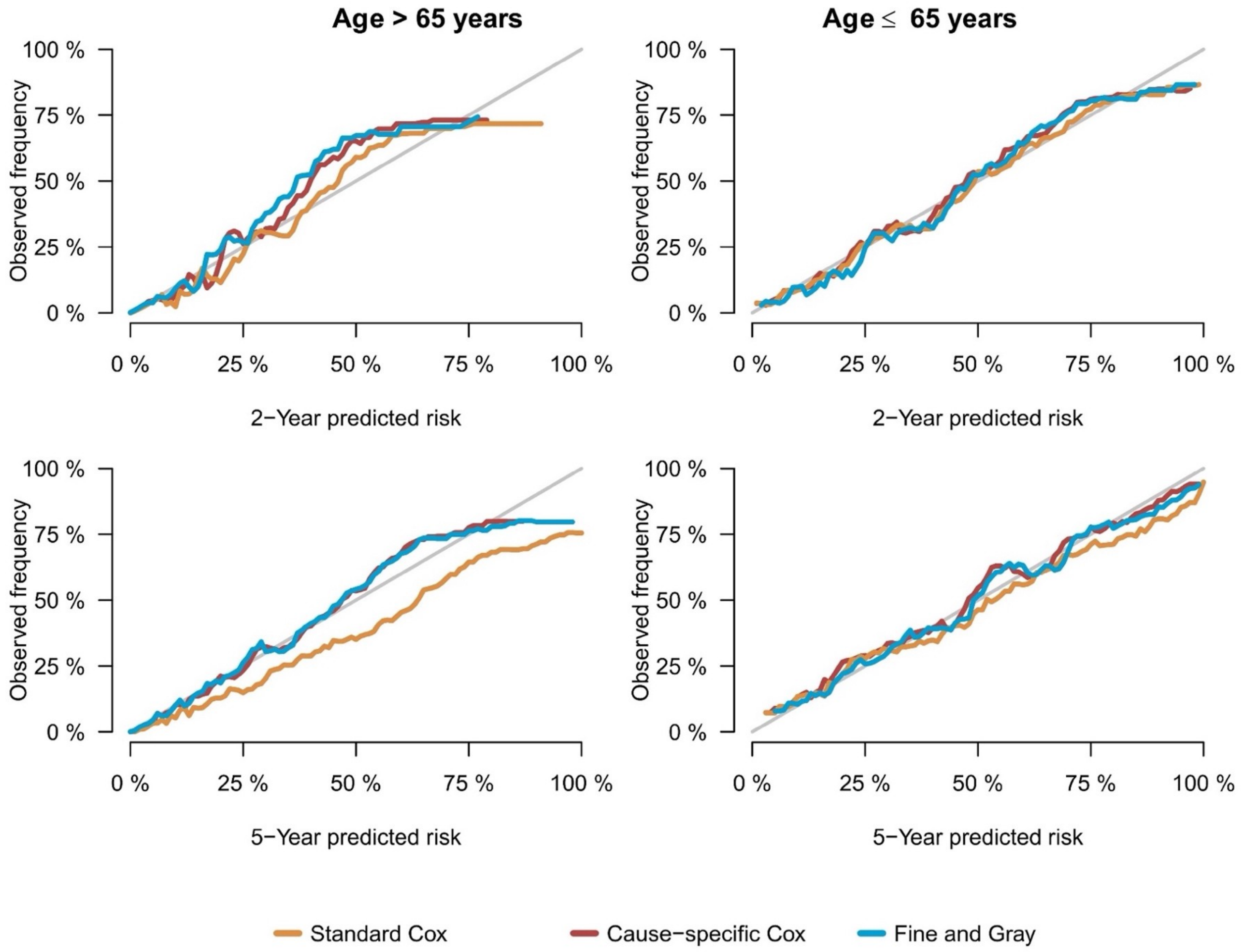

Legend: graphical agreement between observed and predicted risks at years 1-8. In an ideal model, pairs of the observed and predicted risks lie on a 45-degree angle line. Curves falling under the 45-degree angle line indicate that predicted risks overestimate (are higher than) observed risks.

© 2021 Al-Wahsh H et al. *JAMA Network Open.*

eFigure 7: Calibration Plots at 2 and 5 Years by Sex (Development Cohort)

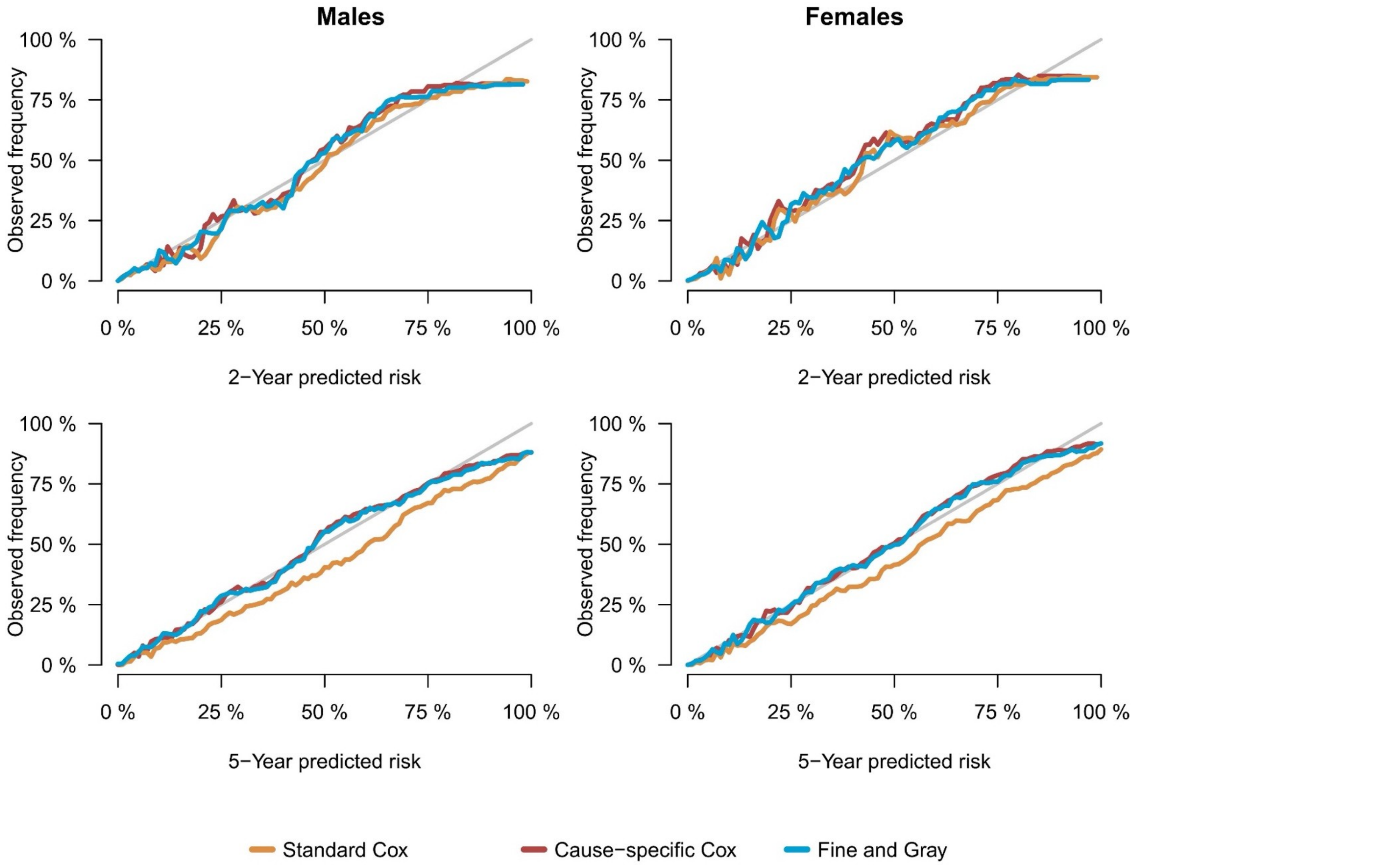

Legend: graphical agreement between observed and predicted risks at years 1-8. In an ideal model, pairs of the observed and predicted risks lie on a 45-degree angle line. Curves falling under the 45-degree angle line indicate that predicted risks overestimate (are higher than) observed risks.

© 2021 Al-Wahsh H et al. *JAMA Network Open.*

eFigure 8: Calibration Plots at 2 and 5 Years by Albumin-Creatinine Ratio (ACR; Development Cohort)

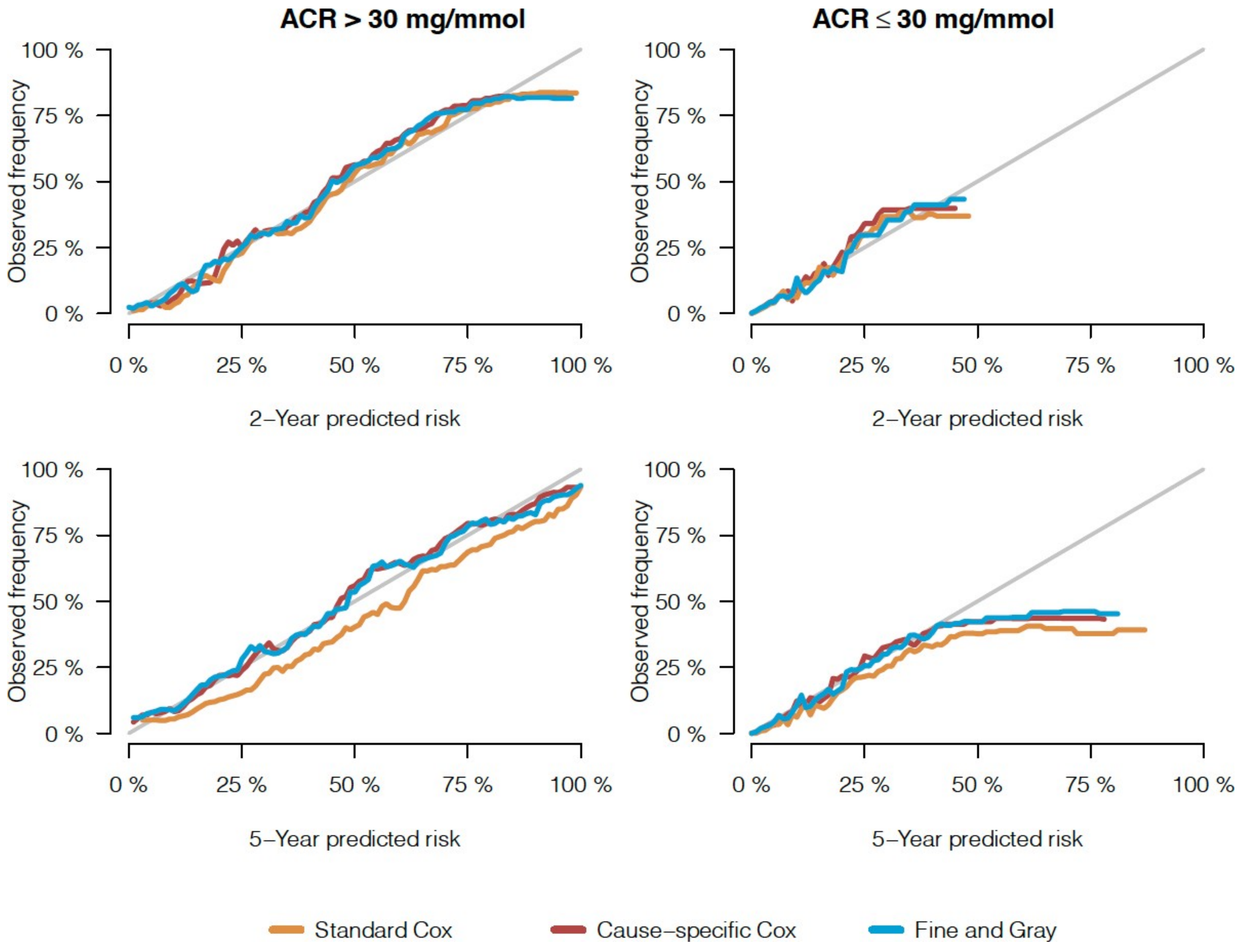

Legend: graphical agreement between observed and predicted risks at years 1-8. In an ideal model, pairs of the observed and predicted risks lie on a 45-degree angle line. Curves falling under the 45-degree angle line indicate that predicted risks overestimate (are higher than) observed risks.

© 2021 Al-Wahsh H et al. *JAMA Network Open.*

eFigure 9. Calibration Plots at 2 and 5 Years by Median Estimated Glomerular Filtration Rate(Development Cohort)

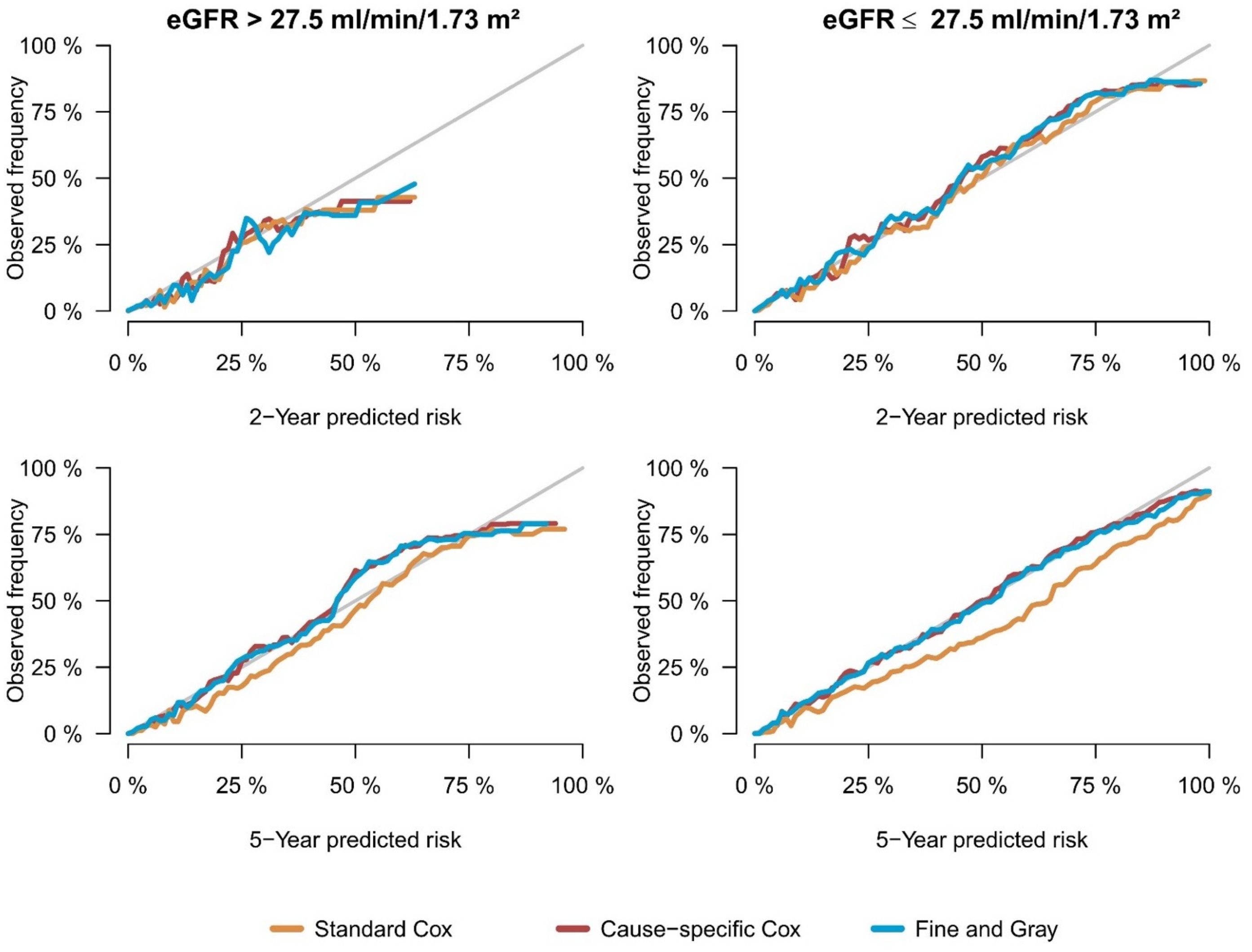

Legend: graphical agreement between observed and predicted risks at years 1-8. In an ideal model, pairs of the observed and predicted risks lie on a 45-degree angle line. Curves falling under the 45-degree angle line indicate that predicted risks overestimate (are higher than) observed risks.

© 2021 Al-Wahsh H et al. *JAMA Network Open.*

eFigure 10. Calibration Plots at 2 and 5 Years by Diabetes (Development Cohort)

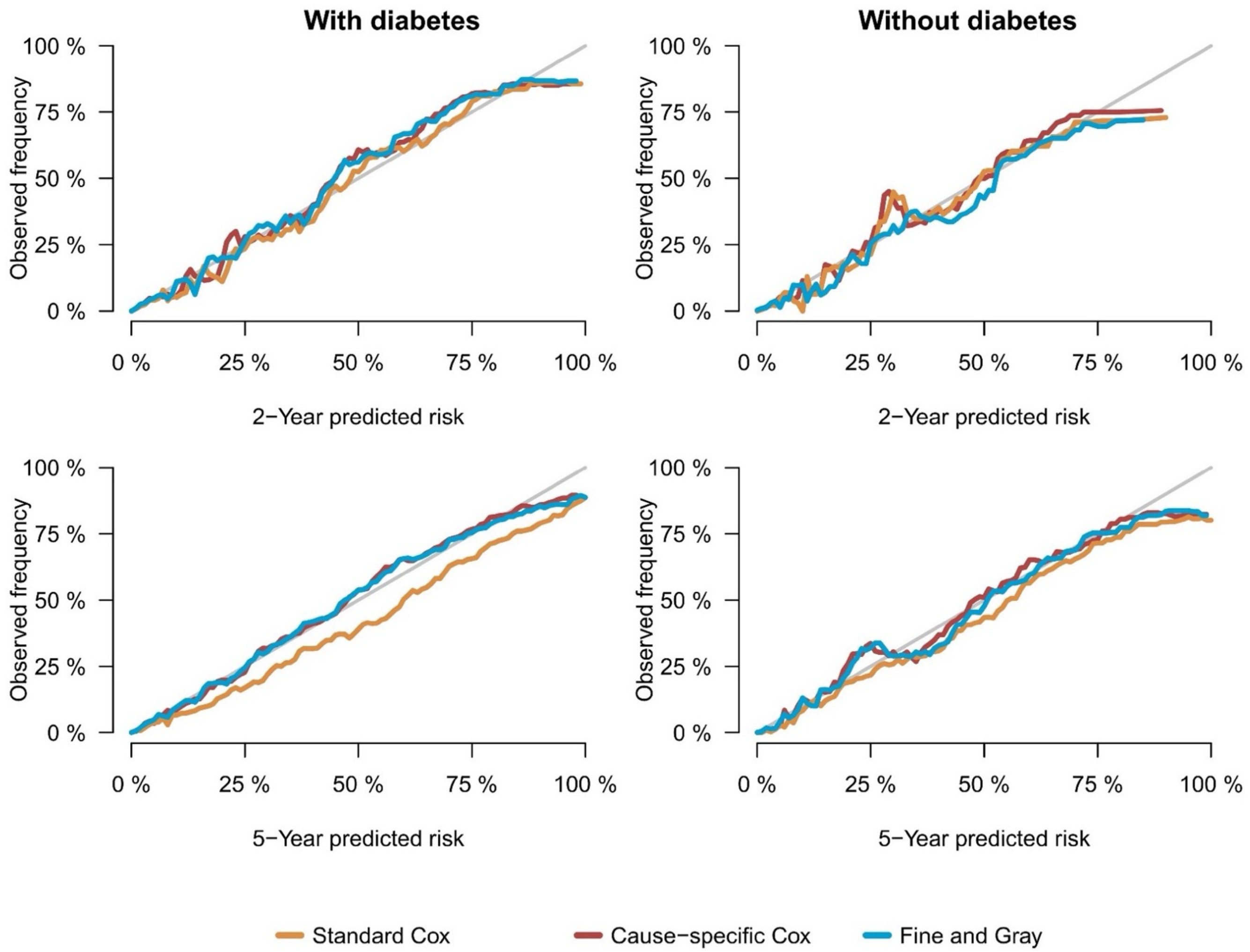

Legend: graphical agreement between observed and predicted risks at years 1-8. In an ideal model, pairs of the observed and predicted risks lie on a 45-degree angle line. Curves falling under the 45-degree angle line indicate that predicted risks overestimate (are higher than) observed risks.

© 2021 Al-Wahsh H et al. *JAMA Network Open.*

eFigure 11. Calibration Plots at 2 and 5 Years by Cardiovascular Disease (Development Cohort)

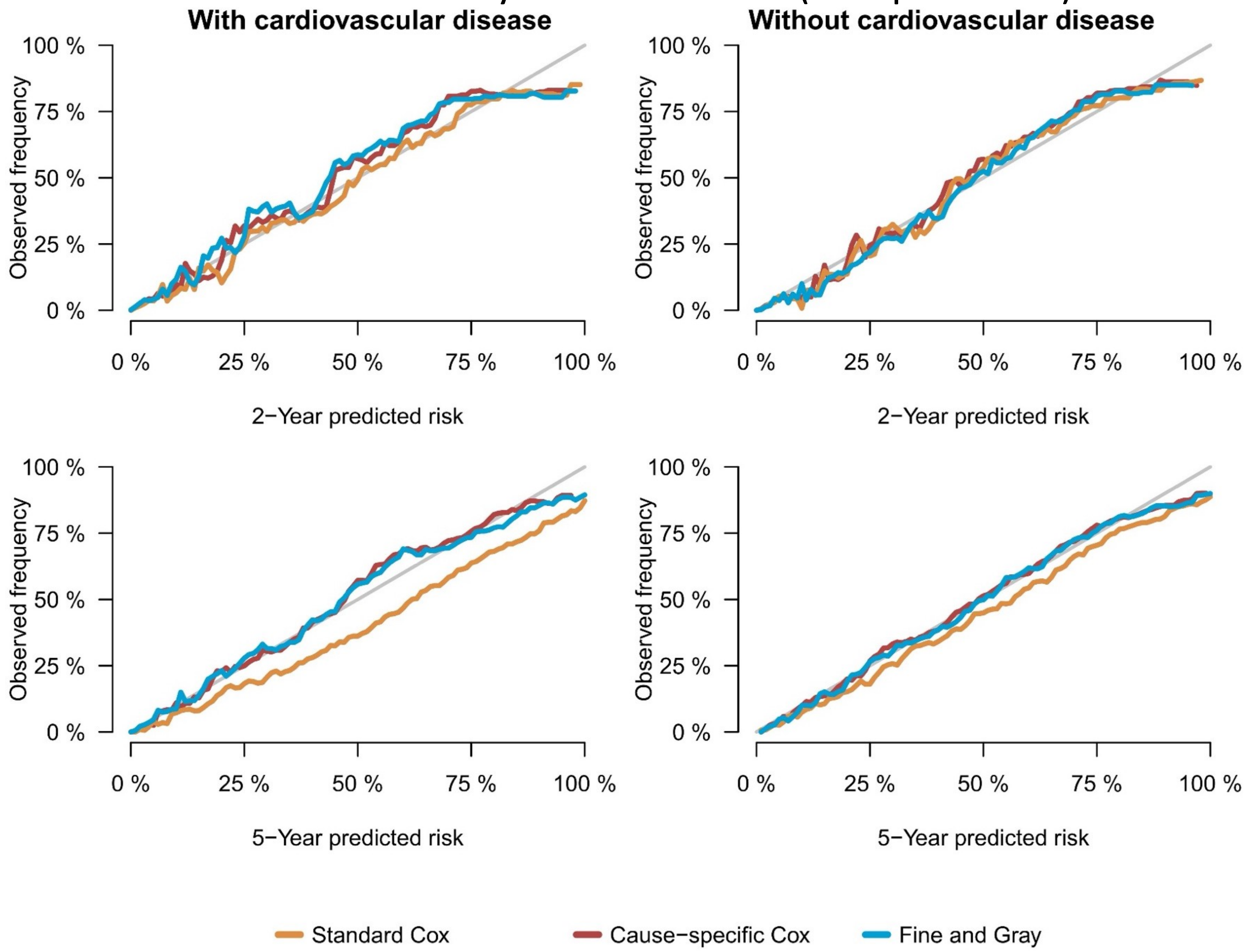

Legend: graphical agreement between observed and predicted risks at years 1-8. In an ideal model, pairs of the observed and predicted risks lie on a 45-degree angle line. Curves falling under the 45-degree angle line indicate that predicted risks overestimate (are higher than) observed risks.

© 2021 Al-Wahsh H et al. *JAMA Network Open.*

eFigure 12. Reclassification Table With Cutoffs at 2 Years of 5% and 15% and at 5 Years of 10% and 20% (Fine and Gray)

Development cohort (N=14,619)

Year 2

Risk category by Fine and Gray	Risk category by standard Cox: <5	≥5 & < 15	≥15
<5	7,727<br>**1.8**	755<br>**4.1**	0
≥ 5 & < 15	250<br>**4.4**	2,556<br>**6.3**	419<br>**12.6**
≥ 15	0	129<br>**10.2**	2,783<br>**36.9**

FG+/SC-	FG-/SC+
1,174 (8.0)	379 (2.6)

Year 5

Risk category by Fine and Gray	Risk category by standard Cox: <10	≥10 & < 20	≥20
<10	6,070<br>**3.2**	1,487<br>**7.5**	60<br>**7.1**
≥ 10 & < 20	81<br>**10.5**	1,428<br>**11.7**	1,034<br>**16.7**
≥ 20	0	35<br>**29.4**	4,424<br>**50.3**

FG+/SC-	FG-/SC+
2,697 (18.4)	0 (0.0)

Validation cohort (N=2,295)

Year 2

Risk category by Fine and Gray	Risk category by standard Cox: <5	≥5 & < 15	≥15
<5	739<br>**3.3**	114<br>**2.6**	0
≥ 5 & < 15	42<br>**35.7**	544<br>**12.4**	94<br>**8.6**
≥ 15	0	34<br>**11.8**	728<br>**34.8**

FG+/SC-	FG-/SC+
208 (9.1)	34 (1.5)

Year 5

Risk category by Fine and Gray	Risk category by standard Cox: <10	≥10 & < 20	≥20
<10	517<br>**5.2**	201<br>**6**	13<br>**89.7**
≥ 10 & < 20	12<br>**37.9**	231<br>**19.6**	192<br>**16**
≥ 20	0	13<br>**23.1**	1,116<br>**52.8**

FG+/SC-	FG-/SC+
406 (17.7)	13 (0.6)

Colour	Meaning
Green	Both models predict correctly
Blue	Neither model predicts correctly
Yellow	Standard Cox correct; cause-specific Cox incorrect
Red	Standard Cox incorrect; cause-specific Cox correct

Legend: Risk (%) was predicted for each member of the development (left) and validation (right) cohort according to the cause-specific Cox and standard Cox models at years 2 and 5 from study entry. People were then assigned to each cell of a 3X3 table corresponding to the combination of the model predictions. Each cell of the 3X3 table includes the number of people (top) and their actual observed risk (crude cumulative incidence function) at 2 or 5 years (bottom, bold). CS-/SC+, total N and
% of people incorrectly classified by cause-specific Cox and correctly classified by standard Cox regression with respect to the actual observed risk; CS+/SC-, total N and
% of people correctly classified by cause-specific Cox and incorrectly classified by standard Cox regression with respect to the actual observed risk.

© 2021 Al-Wahsh H et al. *JAMA Network Open.*

eFigure 13. Reclassification Table With Cutoffs at 2 Years of 5 and 15% and at 5 Years 10% and 20% (Cause-Specific Cox)

Year 2

Development cohort (N=14,619)

Risk category by cause-specific	Risk category by standard Cox: <5	≥5 & < 15	≥15
<5	7,977<br>**1.9**	513<br>**3.5**	0
≥ 5 & < 15	0	2,927<br>**6.4**	307<br>**15.2**
≥ 15	0	0	2,895<br>**35.7**

CS+/SC-	CS-/SC+
513 (3.5)	307 (2.1)

Validation cohort (N=2,295)

Risk category by cause-specific	Risk category by standard Cox: <5	≥5 & < 15	≥15
<5	781<br>**5.1**	75<br>**4**	0
≥ 5 & < 15	0	617<br>**11.6**	80<br>**11.5**
≥ 15	0	0	742<br>**34**

CS+/SC-	CS-/SC+
155 (6.8)	0 (0.0)

Year 5

Development cohort (N=14,619)

Risk category by cause-specific	Risk category by standard Cox: <10	≥10 & < 20	≥20
<10	6,151<br>**3.3**	1,459<br>**8**	36<br>**6.5**
≥ 10 & < 20	0	1,491<br>**11.6**	1,035<br>**16.7**
≥ 20	0	0	4,447<br>**50.1**

CS+/SC-	CS-/SC+
2,530 (17.3)	0 (0.0)

Validation cohort (N=2,295)

Risk category by cause-specific	Risk category by standard Cox: <10	≥10 & < 20	≥20
<10	529<br>**5.9**	203<br>**7**	10<br>**90**
≥ 10 & < 20	0	242<br>**19.1**	204<br>**18.9**
≥ 20	0	0	1,107<br>**52.5**

CS+/SC-	CS-/SC+
407 (17.7)	10 (0.4)

Colour	Meaning	Colour	Meaning
(green)	Both models predict correctly	(yellow)	Standard Cox correct; cause-specific Cox incorrect
(blue)	Neither model predicts correctly	(red)	Standard Cox incorrect; cause-specific Cox correct

Legend: Risk (%) was predicted for each member of the development (left) and validation (right) cohort according to the cause-specific Cox and standard Cox models at years 2 and 5 from study entry. People were then assigned to each cell of a 3X3 table corresponding to the combination of the model predictions. Each cell of the 3X3 table includes the number of people (top) and their actual observed risk (crude cumulative incidence function) at 2 or 5 years (bottom, bold). CS-/SC+, total N and % of people incorrectly classified by cause-specific Cox and correctly classified by standard Cox regression with respect to the actual observed risk; CS+/SC-, total N and % of people correctly classified by cause-specific Cox and incorrectly classified by standard Cox regression with respect to the actual observed risk.

© 2021 Al-Wahsh H et al. *JAMA Network Open.*

eFigure 14. Reclassification Table With Cutoffs at 2 Years of 10 and 40% and at 5 Years of 5 and 50% (Fine and Gray)

Year 2 — Development cohort (N=14,619)

Risk category by Fine and Gray	Risk category by standard Cox: <10	≥10 & < 40	≥40
<10	10,189<br>**2.6**	498<br>**7.7**	0
≥ 10 & < 40	167<br>**4.2**	2,716<br>**19.1**	147<br>**47.2**
≥ 40	0	64<br>**31.5**	838<br>**62.3**

FG+/SC-: 498 (3.4)

FG-/SC+: 378 (2.6)

Year 2 — Validation cohort (N=2,295)

Risk category by Fine and Gray	Risk category by standard Cox: <10	≥10 & < 40	≥40
<10	1,179<br>**6.8**	116<br>**6**	0
≥ 10 & < 40	36<br>**30.6**	671<br>**18.4**	38<br>**44.7**
≥ 40	0	9<br>**22.2**	246<br>**55**

FG+/SC-: 152 (6.6)

FG-/SC+: 47 (2.0)

Year 5 — Development cohort

Risk category by Fine and Gray	Risk category by standard Cox: <5	≥5 & < 50	≥50
<5	2,832<br>**1.8**	1,794<br>**3**	0
≥ 5 & < 50	52<br>**6.1**	7,430<br>**15.3**	576<br>**41**
≥ 50	0	6<br>**20**	1,929<br>**73.3**

FG+/SC-: 2,422 (16.6)

FG-/SC+: 6 (0.0)

Year 5 — Validation cohort

Risk category by Fine and Gray	Risk category by standard Cox: <5	≥5 & < 50	≥50
<5	169<br>**1.8**	176<br>**1.8**	0
≥ 5 & < 50	6<br>**22.2**	1,284<br>**20.1**	142<br>**46.4**
≥ 50	0	3<br>**33.3**	515<br>**73.8**

FG+/SC-: 324 (14.1)

FG-/SC+: 3 (0.1)

Colour	Meaning
Green	Both models predict correctly
Blue	Neither model predicts correctly
Yellow	Standard Cox correct; Fine and Gray incorrect
Red	Standard Cox incorrect; Fine and Gray correct

Legend: Risk (%) was predicted for each member of the development (left) and validation (right) cohort according to the cause-specific Cox and standard Cox models at years 2 and 5 from study entry. People were then assigned to each cell of a 3X3 table corresponding to the combination of the model predictions. Each cell of the 3X3 table includes the number of people (top) and their actual observed risk (crude cumulative incidence function) at 2 or 5 years (bottom, bold). CS-/SC+, total N and % of people incorrectly classified by cause-specific Cox and correctly classified by standard Cox regression with respect to the actual observed risk; CS+/SC-, total N and % of people correctly classified by cause-specific Cox and incorrectly classified by standard Cox regression with respect to the actual observed risk.

© 2021 Al-Wahsh H et al. *JAMA Network Open.*

eFigure 15. Reclassification Table With Cutoffs at 2 Years of 10% and 40% and at 5 Years of 5% and 50% (Cause-Specific Cox)

Development cohort (N=14,619) — Year 2

Risk category by standard Cox (columns); Risk category by cause-specific (rows)

	<10	≥10 & < 40	≥40
<10	10,356<br>**2.6**	337<br>**6.6**	0
≥ 10 & < 40	0	2,941<br>**18.9**	108<br>**43.6**
≥ 40	0	0	877<br>**62**

CS+/SC-: 337 (2.3)

CS-/SC+: 108 (0.7)

Validation cohort (N=2,295) — Year 2

Risk category by standard Cox (columns); Risk category by cause-specific (rows)

	<10	≥10 & < 40	≥40
<10	1215<br>**7.5**	86<br>**9.3**	0
≥ 10 & < 40	0	710<br>**17.6**	35<br>**42.9**
≥ 40	0	0	249<br>**55.1**

CS+/SC-: 86 (3.7)

CS-/SC+: 35 (1.5)

Development cohort (N=14,619) — Year 5

Risk category by standard Cox (columns); Risk category by cause-specific (rows)

	<5	≥5 & < 50	≥50
<5	2,884<br>**1.9**	1,659<br>**3.2**	0
≥ 5 & < 50	0	7,571<br>**15**	602<br>**41.7**
≥ 50	0	0	1,903<br>**73.5**

CS+/SC-: 2,261 (15.5)

CS-/SC+: 0 (0.0)

Validation cohort (N=2,295) — Year 5

Risk category by standard Cox (columns); Risk category by cause-specific (rows)

	<5	≥5 & < 50	≥50
<5	175<br>**2.4**	171<br>**1.2**	0
≥ 5 & < 50	0	1292<br>**20.1**	148<br>**41.7**
≥ 50	0	0	509<br>**75.6**

CS+/SC-: 319 (13.9)

CS-/SC+: 0 (0.0)

Colour	Meaning	Colour	Meaning
(green)	Both models predict correctly	(yellow)	Standard Cox correct; cause-specific Cox incorrect
(blue)	Neither model predicts correctly	(pink)	Standard Cox incorrect; cause-specific Cox correct

Legend: Risk (%) was predicted for each member of the development (left) and validation (right) cohort according to the cause-specific Cox and standard Cox models at years 2 and 5 from study entry. People were then assigned to each cell of a 3X3 table corresponding to the combination of the model predictions. Each cell of the 3X3 table includes the number of people (top) and their actual observed risk (crude cumulative incidence function) at 2 or 5 years (bottom, bold). CS-/SC+, total N and % of people incorrectly classified by cause-specific Cox and correctly classified by standard Cox regression with respect to the actual observed risk; CS+/SC-, total N and % of people correctly classified by cause-specific Cox and incorrectly classified by standard Cox regression with respect to the actual observed risk.

© 2021 Al-Wahsh H et al. *JAMA Network Open.*

eFigure 16. Calibration Plots at 2 and 5 Years by Cohort Entry on or Before vs After the Median Index Date (Development Cohort)

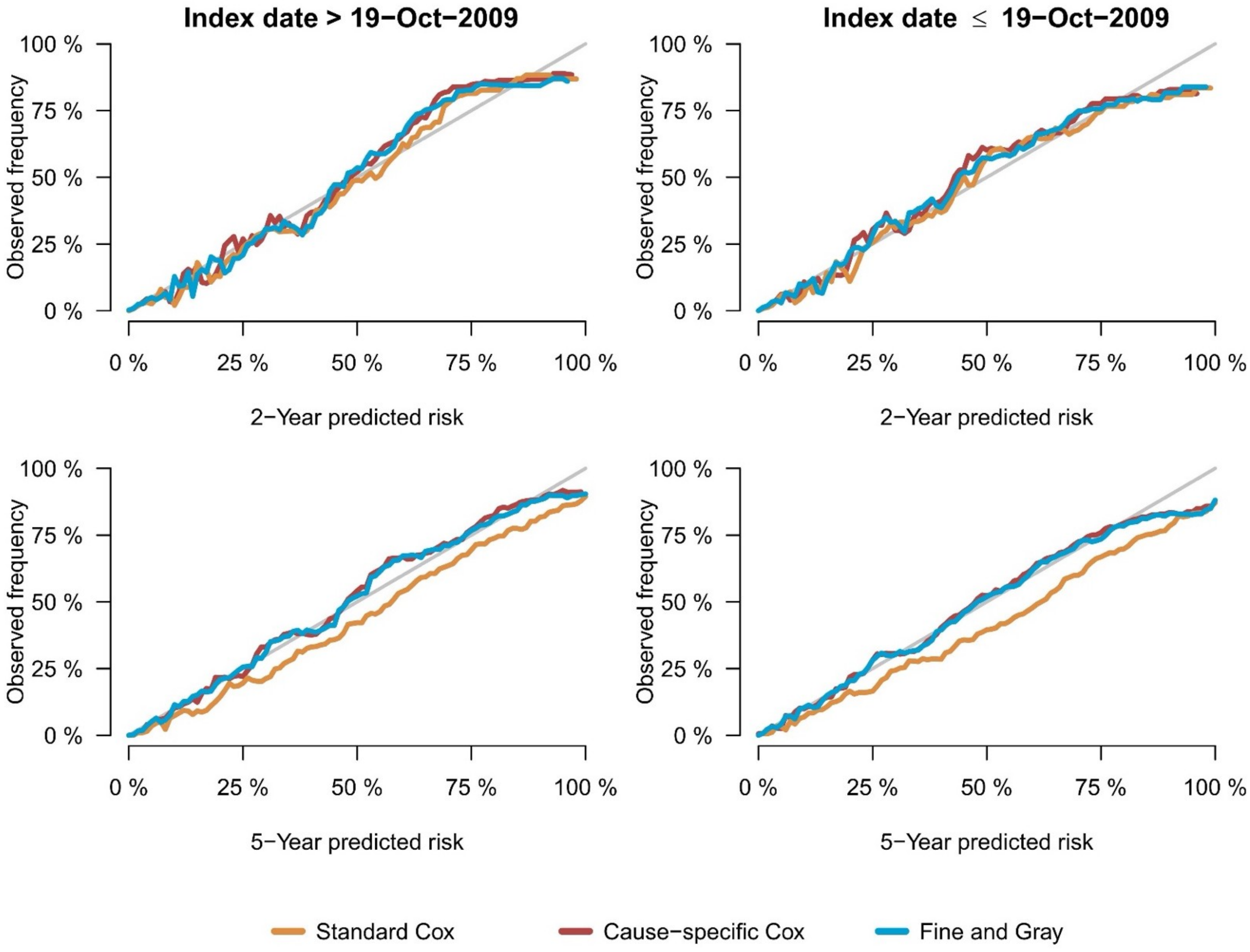

Legend: graphical agreement between observed and predicted risks at years 1-8. In an ideal model, pairs of the observed and predicted risks lie on a 45-degree angle line. Curves falling under the 45-degree angle line indicate that predicted risks overestimate (are higher than) observed risks.

© 2021 Al-Wahsh H et al. *JAMA Network Open.*

eFigure 17: Calibration Plots at 1, 3, 5, and 7 Years (Sensitivity Analysis)

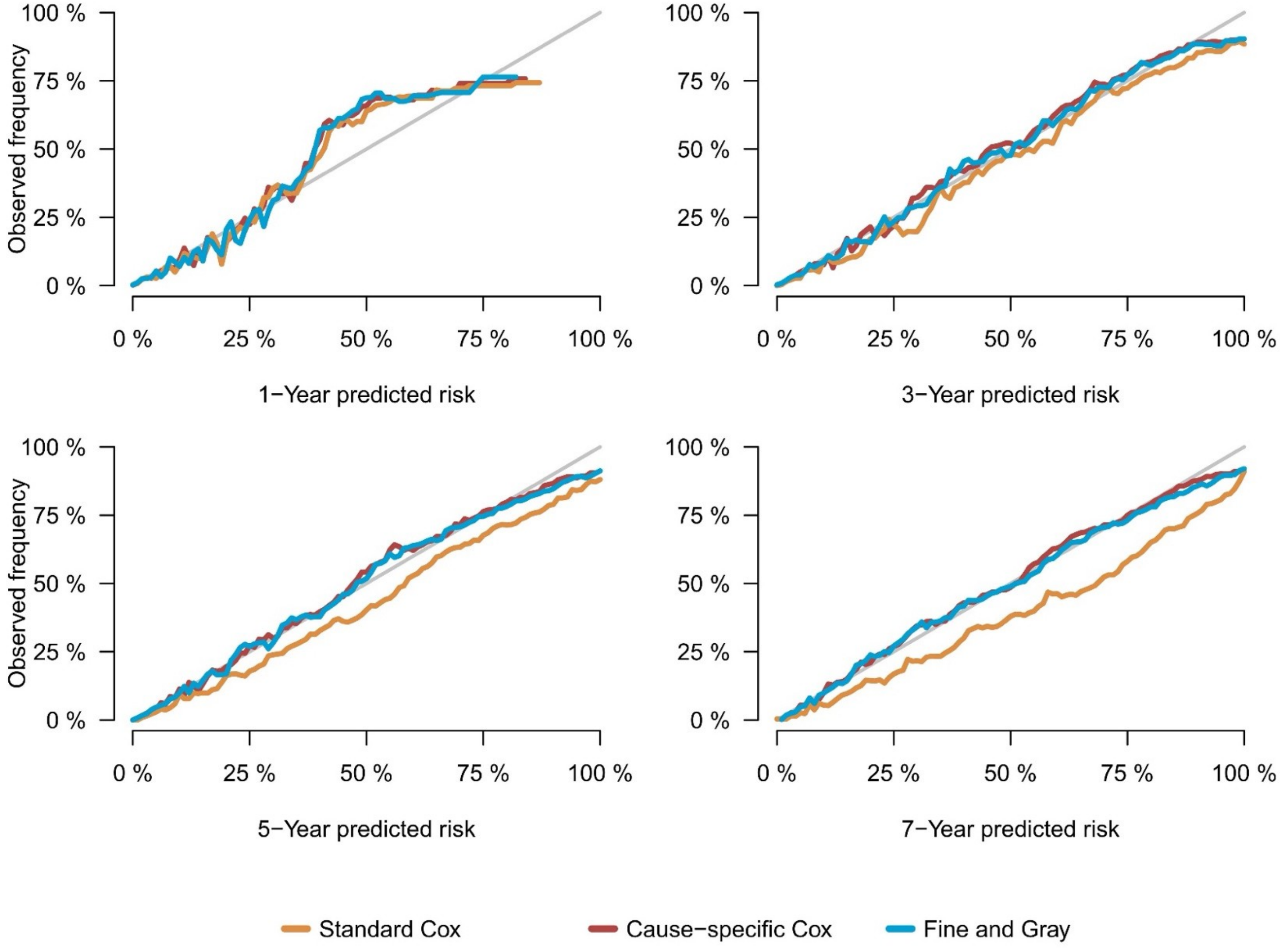

Legend: In this sensitivity analysis kidney failure was defined as initiation of renal replacement or occurrence of sustained eGFR <10 ml/min/1.73 m^2, instead of initiation of renal replacement therapy or moving average eGFR <10 ml/min/1.73 m^2 (3,126 kidney failure events and 6,621 death events; person-year at risk: 53,487).

© 2021 Al-Wahsh H et al. *JAMA Network Open.*

eFigure 18: Calibration Plots at 2, 4, 6, and 8 Years (Sensitivity Analysis)

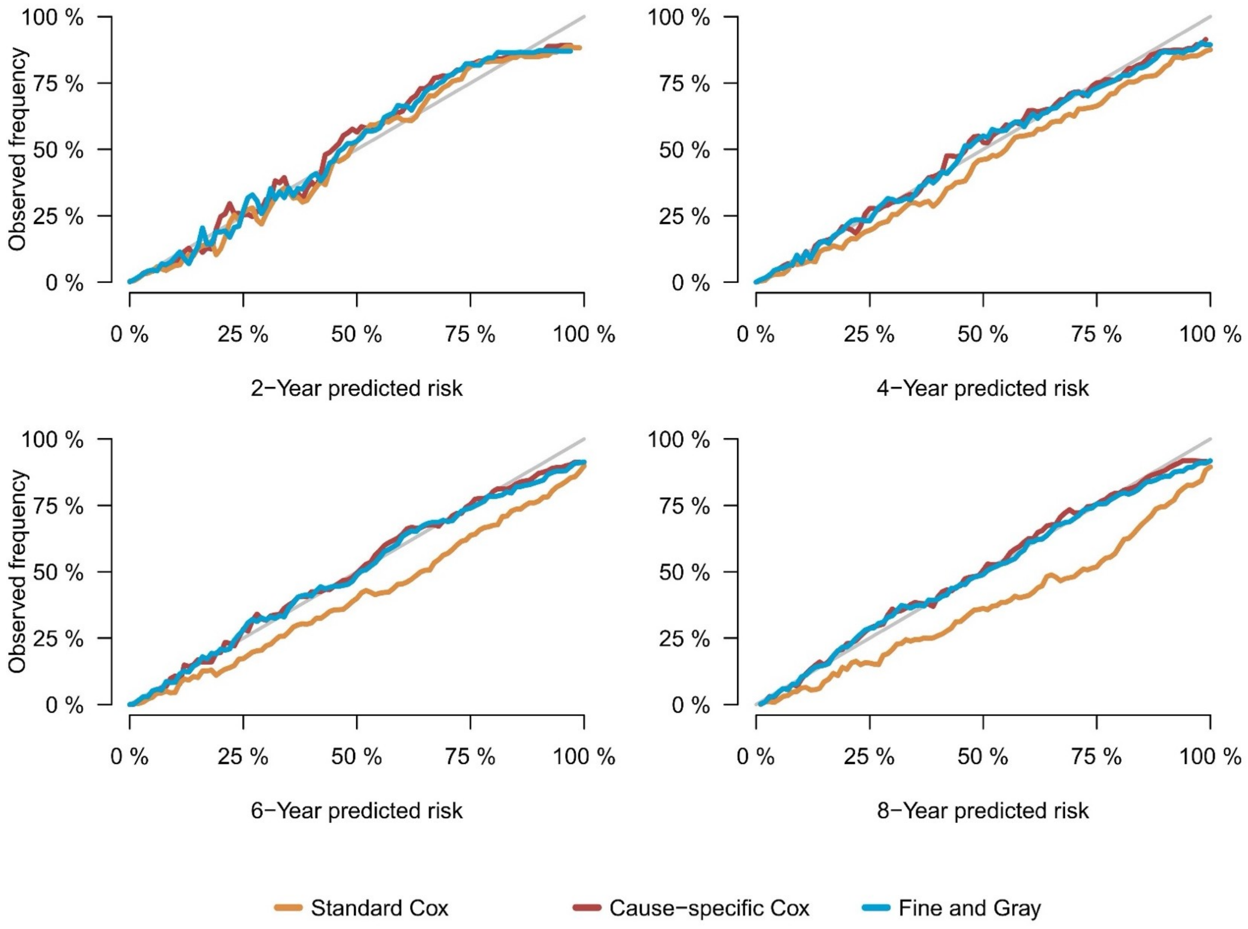

Legend: In this sensitivity analysis kidney failure was defined as initiation of renal replacement or occurrence of sustained eGFR <10 ml/min/1.73 m^2, instead of initiation of renal replacement therapy or moving average eGFR <10 ml/min/1.73 m^2 (3,126 kidney failureevents and 6,621 death events; person-year at risk: 53,487).

© 2021 Al-Wahsh H et al. *JAMA Network Open.*

References

1. Tonelli M, Wiebe N, Fortin M, et al. Methods for identifying 30 chronic conditions: Application to administrative data Healthcare Information Systems. *BMC Med Inform Decis Mak*. Published online 2015:15-31.

© 2021 Al-Wahsh H et al. *JAMA Network Open.*
